# ATRA-Induced Cellular Differentiation and CD38 Expression Inhibits Acquisition of BCR-ABL Mutations for CML Acquired Resistance

**DOI:** 10.1371/journal.pgen.1004414

**Published:** 2014-06-26

**Authors:** Zhiqiang Wang, Zheng Liu, Xiwei Wu, Su Chu, Jinhui Wang, Hongfeng Yuan, Mendel Roth, Yate-Ching Yuan, Ravi Bhatia, WenYong Chen

**Affiliations:** 1 Department of Cancer Biology, Beckman Research Institute, City of Hope, Duarte, California, United States of America; 2 Department of Molecular Medicine, Beckman Research Institute, City of Hope, Duarte, California, United States of America; 3 Division of Hematopoietic Stem Cell and Leukemia Research, Beckman Research Institute, City of Hope, Duarte, California, United States of America; University of Washington, United States of America

## Abstract

Acquired resistance through genetic mutations is a major obstacle in targeted cancer therapy, but the underlying mechanisms are poorly understood. Here we studied mechanisms of acquired resistance of chronic myeloid leukemia (CML) to tyrosine kinase inhibitors (TKIs) by examining genome-wide gene expression changes in KCL-22 CML cells versus their resistant KCL-22M cells that acquire T315I BCR-ABL mutation following TKI exposure. Although T315I BCR-ABL is sufficient to confer resistance to TKIs in CML cells, surprisingly we found that multiple drug resistance pathways were activated in KCL-22M cells along with reduced expression of a set of myeloid differentiation genes. Forced myeloid differentiation by all-trans-retinoic acid (ATRA) effectively blocked acquisition of BCR-ABL mutations and resistance to the TKIs imatinib, nilotinib or dasatinib in our previously described in vitro models of acquired TKI resistance. ATRA induced robust expression of CD38, a cell surface marker and cellular NADase. High levels of CD38 reduced intracellular nicotinamide adenine dinucleotide (NAD^+^) levels and blocked acquired resistance by inhibiting the activity of the NAD^+^-dependent SIRT1 deacetylase that we have previously shown to promote resistance in CML cells by facilitating error-prone DNA damage repair. Consequently, ATRA treatment decreased DNA damage repair and suppressed acquisition of BCR-ABL mutations. This study sheds novel insight into mechanisms underlying acquired resistance in CML, and suggests potential benefit of combining ATRA with TKIs in treating CML, particularly in advanced phases.

## Author Summary

Acquired resistance through genetic mutations is a major mechanism for cancer drug resistance and accounts for the short life of targeted therapy in several types of human cancer. Mechanistically, however, very little is understood about how resistant mutations are actually acquired during cancer therapy. In this manuscript, we used chronic myelogenous leukemia (CML) as a disease model and showed that mutation acquisition process is accompanied by global genome transcriptional reprogramming and reduction of cellular differentiation status. Forced cell differentiation by all-trans retinoic acid (ATRA) potently blocks acquisition of genetic mutations and CML acquired resistance. ATRA effect is mediated, in part, through stimulating CD38 gene expression, which reduces cellular cofactor nicotinamide adenine dinucleotide (NAD^+^) content and thus the activity of NAD^+^-dependent protein deacetylase SIRT1 that promotes error-prone DNA damage repair and mutagenesis. Our findings provide novel insight of mutation acquisition process during targeted therapy for CML. This study has translational implication in clinical treatment of CML, and perhaps other malignancies, by combining a differentiation agent to overcome mutation-mediated drug resistance if possible.

## Introduction

Chronic myeloid leukemia (CML) is a myeloproliferative disease resulting from the clonal hematopoietic stem cell disorder that is caused by the transformation of oncogenic breakpoint cluster region-Abelson (BCR-ABL) fusion gene [1]. Typically, CML progresses from chronic phase (CP) to accelerated phase (AP) then into blast crisis (BC), which can be distinguished by the number and maturation of leukocytes. Treatment with imatinib mesylate (IM), a BCR-ABL tyrosine kinase inhibitor, can effectively yield a durable complete cytogenetic response in CP patients and the drug is widely used as the first-line therapy for most CML patients [2]. However, residual leukemia cells persist in nearly all patients that may account for the disease recurrence if the treatment is discontinued [3], [4].

The emergence of point mutations in the BCR-ABL kinase domain is a major cause of imatinib resistance in CML patients, especially in AP and BC [5], [6]. These acquired mutations may alter kinase domain structure and impair drug binding affinity. The second generation tyrosine kinase inhibitors nilotinib and dasatinib show much more potent activity against BCR-ABL and most mutants, but some kinase domain mutations, especially T315I, are still resistant to these drugs [7]–[9]. Although TKIs such as Ponatinib [10], with activity against the T315I mutation, have been developed, their application to CML therapy has been limited by concerns regarding toxicity. In addition, highly resistant compound mutations appear to be an emerging problem. Therefore, more rational therapeutic strategies still need to be developed to overcome the problem of TKI resistance.

We have recently described a novel model of acquired resistance in CML using the blast crisis CML cell line KCL-22 [11]. In this model, the cells initially undergo apoptosis upon treatment with therapeutically effective doses of imatinib, but then re-grow within two weeks by development of resistance through T315I BCR-ABL mutation [11]. This model provides a very useful tool to study molecular mechanisms of acquisition of BCR-ABL mutations from its native chromatin locus. We have shown that the native BCR-ABL locus has nearly ten times higher mutagenesis potential than randomly integrated BCR-ABL cDNA in the same cells, suggesting the likely influence of the genetic instability or epigenetic deregulation from the translocation locus [11]. We have identified a key epigenetic regulator sirtuin 1 (SIRT1), a NAD^+^-dependent protein lysine deacetylase, that promotes BCR-ABL mutagenesis through stimulating error-prone DNA damage repair [12]. Using this model, we have also demonstrated that mitotic kinase Aurora A plays a crucial role in facilitating newly emerging mutant cells to pass through initial mitotic crisis, leading to eventual relapse [13], which is in line with our proposal that acquisition of BCR-ABL mutations is a multi-step process [14].

To further delineate the mechanisms of BCR-ABL mutation acquisition, in the present study, we carried out microarray gene expression analysis of KCL-22 cells versus KCL-22M cells that are derived from KCL-22 cells after acquiring T315I mutation upon IM treatment [11]. Interestingly, we found that KCL-22M cells exhibit genome-wide gene expression changes in several pathways that may increase drug resistance, in addition to the presence of T315I mutation. Furthermore, altered expression of several myeloid differentiation genes indicates that KCL-22M cells are in a less differentiated state than KCL-22 cells. By exome sequencing, we found that the altered gene expression patterns are not obviously correlated with genome-wide codon mutations in KCL-22M cells. Inspired by the microarray analysis, we tested the effect of induced differentiation by all-trans retinoic acid (ATRA) that is frequently used in clinical treatment of acute promyelocytic leukemia (APL) [15], [16]. We found that ATRA potently blocks acquisition of BCR-ABL mutations (T315I, Y253H and E255K) and regrowth of CML cells on imatinib treatment. ATRA executes its effect in part through activating expression of CD38, a major NADase in mammalian cells [17], which reduces cellular NAD^+^ levels and consequently inhibits the activity of SIRT1. Consistently, we found that CD38 expression levels are significantly reduced as CML progresses from chronic phase to blast crisis, which may support high activity of SIRT1 in the late phase of the disease. This study suggests a novel role of cellular differentiation status and CD38 expression on acquisition of BCR-ABL mutations, and the potential therapeutic use of ATRA to inhibit CML acquired resistance to tyrosine kinase inhibitors.

## Results

### Genome-wide changes of gene expression accompanied BCR-ABL mutation acquisition

**Figure 1:**
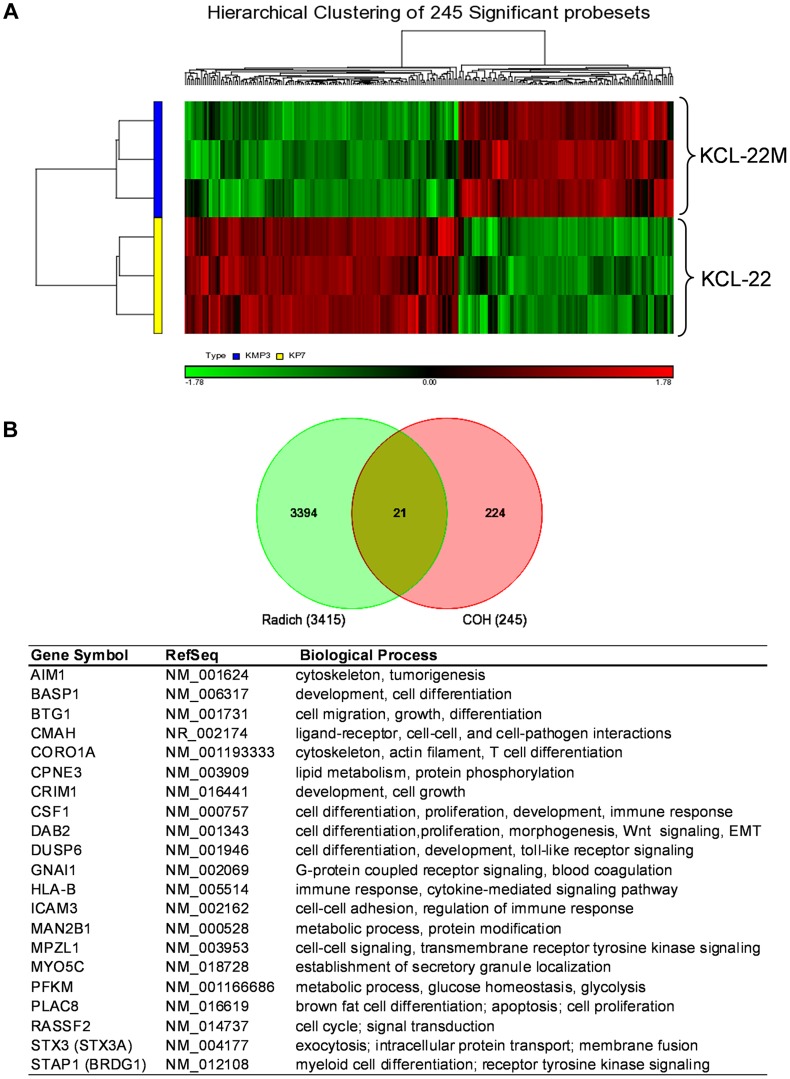
Microarray gene expression analysis of KCL-22 vs KCL-22M cells. (A) Hierarchical clustering of KCL-22M and KCL-22 samples based on the 245 significant probe sets. Clearly, KCL-22M and KCL-22 formed two separate clusters. (B) Venn diagram analysis of 245 significant probe sets of this study versus the published 3415 CML “phase reporter” gene sets from Radich J.P et al. (PNAS 2006, 103: 2794–2799). Details of 21 overlapping genes were listed.

We carried out microarray gene expression analysis of KCL-22M vs KCL-22 cells using Affymetrix gene chips. Triplicate RNA samples of each cell type were harvested and used for the study. Principal component analysis showed clear separation between KCL-22M and KCL-22 samples, indicting distinct biological groups (Figure S1A). Expression of the vast majority of the genes from these two cell types was indistinguishable from the volcano plot (Figure S1B), consistent with the fact these two cell types have only small biological difference except for IM resistance [11]. However, we identified 245 probe sets with expression change bigger than 1.5 fold and p value<0.05, which formed separate hierarchical clusters (Figure 1 and Table S1). We compared these probe sets with the largest published microarray data of primary human CML samples by Radich et al [18] who showed signature gene expression changes as CML progresses from chronic to accelerated and blast crisis phases, as well as relapsed CML. By Venn diagram analysis, 21 probe sets were found overlapping with Radich's 3415 “phase reporter” gene sets (Figure 1B). Since only 160 among the 245 probe sets were annotated (Table S1), the overlapping genes represented 13% of our annotated probe sets. Notably, among these 21 genes, many of them are involved in regulation of differentiation and development (Figure 1B).

**Figure 2:**
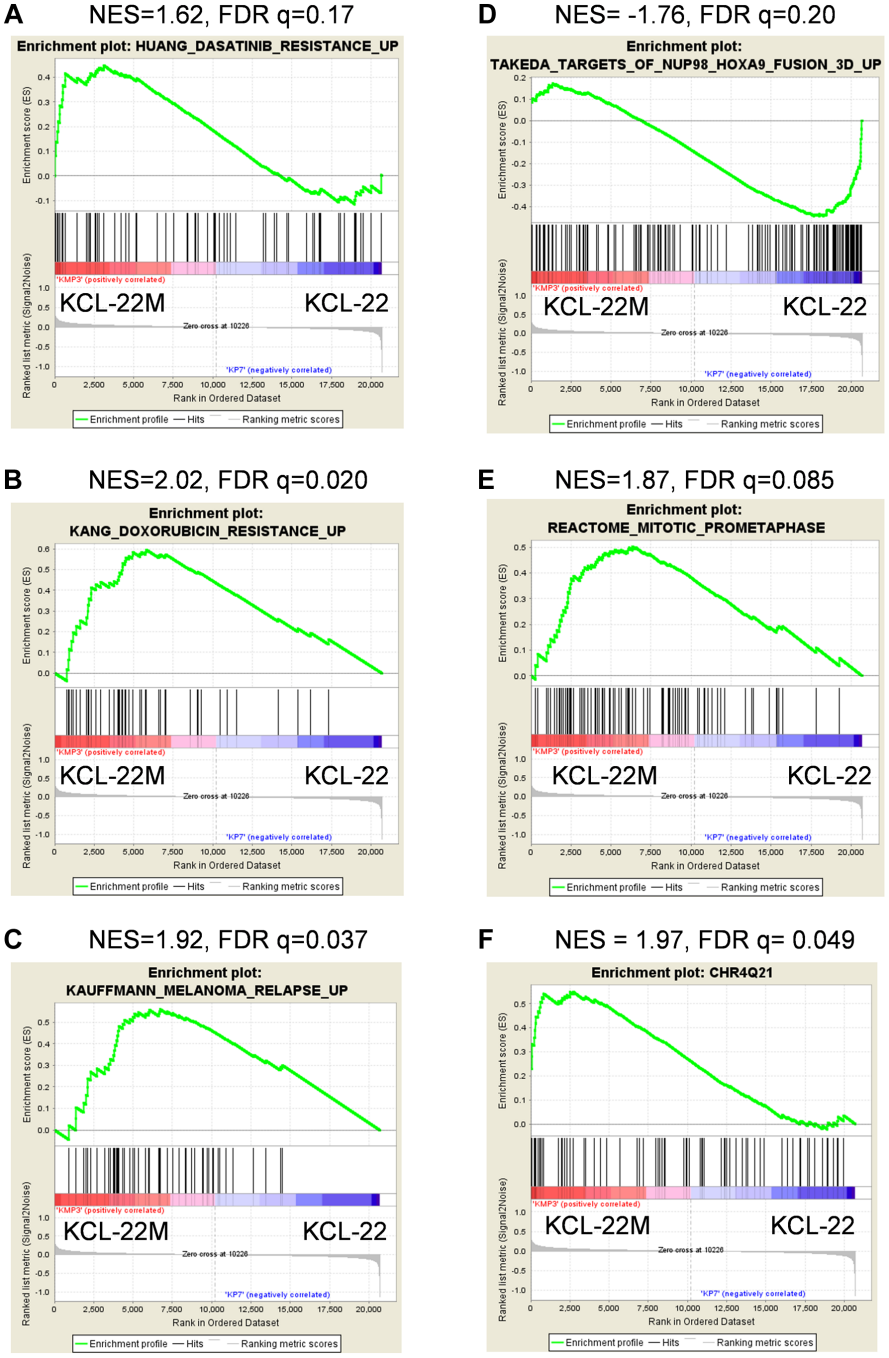
Enrichment plots for significant pathways identified by GSEA. Enrichment plots related with cancer drug resistance and relapse (A–C), CML cell transformation (D), and mitotic cell regulation (E). Plots A–D were identified by C2CGP and E by C2CP gene sets. The enrichment plot for the most significant regional chromosomal change on the Chr4Q21 by C1 positional gene set analysis was shown in (F).

Using gene set enrichment analysis (GSEA) [19], we found that KCL-22M cells were significantly enriched for dasatinib resistance pathway genes [20] and doxorubicin/cisplatin resistance genes [21] found in solid tumors as well as genes activated in relapsed melanoma cells [22] (Figure 2A–C). These changes were surprising given that the T315I BCR-ABL mutant is sufficient to confer resistance of KCL-22M cells to imatinib [11] and it would seem that activation of these resistance pathways would be unnecessary. The array data suggest that genome-wide transcriptome changes are likely coupled with BCR-ABL mutation acquisition process that occurs in KCL-22 cells in response to imatinib treatment, and which continues to be present in KCL-22M cells. This is in line with our previous finding that mutations in KCL-22M cells are acquired *de novo* instead of by simple outgrowth of cells with spontaneously acquired T315I mutation [11], [14].

GSEA showed that KCL-22M cells had reduced expression of genes upregulated by NUP98/HOXA9 (Figure 2D), a fusion gene that blocks differentiation and promotes CML transformation [23]–[25], consistent with our previous finding that KCL-22M cells have reduced soft agar colony formation and transformation capability than KCL-22 cells [11]. Expression of mitotic genes was also significantly enriched in KCL-22M cells (Figure 2E), consistent with the increased G2/M cell fractions in KCL-22M cells and their attempt to overcome mitotic crisis during the early stage of mutation acquisition [11], [13]. Changes of several cell cycle related genes were also found in a previous study of primary CD34^+^ CML progenitor cells [26], Figure S2. Together, our microarray data faithfully recapitulate the experimental observations.

By spectral karyotyping analysis, we previously showed that there are no gross chromosomal changes in KCL-22M cells compared to KCL-22 cells [11]. Interestingly, by analysis of KCL-22M>KCL-22 cells using the GSEA positional gene set analysis that allows identification of regional chromosomal changes (indel, amplification & epigenetic silencing) affecting gene expression [19], we found 20 loci in KCL-22M cells with significant changes (FDR<0.25, p<0.05, Table S2), with the Chr4Q21 region having the most significant gene enrichment in KCL-22M cells (Figure 2F). No significant loci were identified in the complimentary analysis (KCL-22>KCL-22M) as expected. These data indicate the possibility of some small chromosomal changes or epigenetic alteration accompanying with acquisition of BCR-ABL mutations.

**Figure 3:**
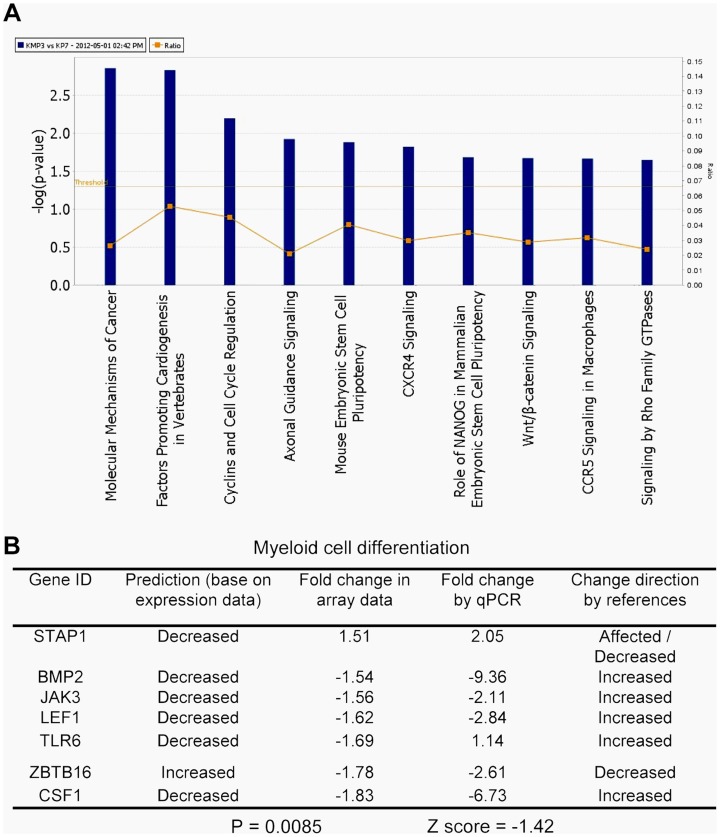
Key pathway changes identified by ingenuity pathway analysis. (A) The top ten altered canonical signaling pathways. Note that three pathways were related with stem cell functions: embryonic stem cell pluripotency, Nanog, and Wnt. (B) Alteration of myeloid differentiation pathway. The expression changes of all seven genes by array analysis were validated by real time quantitative PCR. “Change direction by references” was compiled by the software for the impact of expression of a gene on myeloid differentiation according to literature. “Prediction” indicated potential impact on differentiation based on array gene expression data. STAP1 gene was re-annotated as Affected/Decreased under “Change direction by references” according to current literature, but the Z score and p value were based on STAP1 annotated as “Affected” in the software.

We then used Ingenuity Pathways Analysis to search for additional pathway changes in KCL-22M cells. The leading altered signaling pathway was “molecular mechanisms of cancer” (Figure 3A). Among top ten canonical pathways, three were related with the increased stem cell functions including Wnt/β-catenin signaling (Figure 3A). Hematopoietic development is one of the lead functional changes (Figure S3). Specifically, expression of 6 out of 7 myeloid cell differentiation pathway genes was significantly changed in a way to indicate the less differentiated status of KCL-22M cells than KCL-22 cells, and the expression changes of these genes were validated by real time PCR analysis (Figure 3B). Both increased Wnt/β-catenin signaling and reduced myeloid differentiation are also among the top pathways for CML progression towards advanced disease in the Radich's study [18]. Two myeloid differentiation genes (STAP1 and CSF1) were among the 21 overlapping genes mentioned above (Figure 1B). Intriguingly, it is shown that chronic CML patients who relapsed after initially successful imatinib treatment (many with acquisition of BCR-ABL mutations) demonstrate gene expression signatures more similar to advanced disease than chronic phase [18], and may have reduced differentiation as well. Therefore, our finding that BCR-ABL mutation acquisition process may be accompanied by reduced differentiation state of KCL-22 cells is clinically relevant.

### Genome-wide genetic alterations in KCL-22M cells

**Figure 4:**
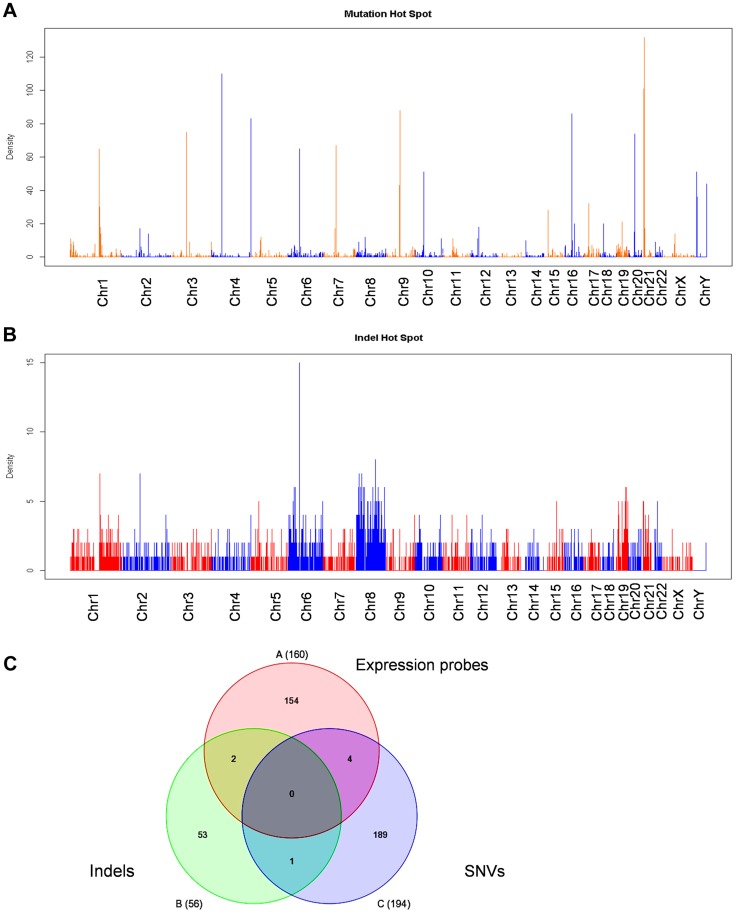
Exome sequencing analysis of KCL-22M vs KCL-22 cells. (A, B) Chromosomal distribution of point mutation (A) and indel (B) hot spots identified specifically in KCL-22M cells. The counts of point mutation or indel events in 1 Mb windows along each chromosome were drawn. (C) Venn diagram analysis of significant gene expression probe sets from Figure 1A, point mutations and indels in coding exons. The numbers included 160 annotated probes from the 245 expression probe sets, 194 unique genes for a total of 208 point mutations in coding exons, and 56 accession numbers for a total of 33 unique genes bearing frame-shift indels in coding exons. None of 7 overlapping genes were on chromosome 4.

We next examined whether there are genetic alterations acquired in KCL-22M cells that may cause genome-wide gene expression changes. We performed Solexa exome capture sequencing of KCL-22 and KCL-22M cells, and generated about 130 million paired-end reads for each cell type. Over 98% of the sequences were aligned to human reference genome hg19, covering more than 80% exons with >20× coverage. Single nucleotide variants (SNVs) identified in both cell types had >97% dbSNP concordance, and the overall transition/transversion ratio was the same at 1.90 in both cell types. After removing SNVs from KCL-22 cells, 3260 point mutations were identified specifically in KCL-22M cells. These mutations distributed throughout the chromosomes with a few mutation hot spots on several chromosomes (Figure 4A). Among these mutations, only 208 were on coding exons of 194 genes, with a few genes bearing more than one mutation (Table S3). T315I mutation was identified as the only mutation on the ABL codons, consistent with our previous finding that T315I mutation is the sole mutation of BCR-ABL in KCL-22M cells [11]. No mutations were found in the known major epigenome regulators.

By copy number variant analysis, we did not detect large amplification or deletion in KCL-22M cells compared to KCL-22 (data not shown), in line with our previous spectral karyotyping analysis [11]. However, we identified 2206 small indels including 1643 deletions and 563 insertions specifically in KCL-22M cells, and they were distributed throughout chromosomes (Figure 4B). The small indels caused frameshift in the codons of 33 genes (Table S4). Although Chr4Q21 was a most significant region for gene expression change, chromosome 4 carried mutation but not indel hot spots (Figure 4A,B). When the 245 significant microarray probe sets were compared to genes with mutations and indels on coding exons, poor overlap was found (Figure 4C), suggesting that global gene expression changes and mutations/indels may occur in parallel or separately. In line with this finding, we previously showed that BCR-ABL expression levels did not change in spite of its acquisition of T315I mutation in KCL-22M cells [11]. It is possible that global gene expression changes may be a result of certain yet-be-identified epigenome reprogramming process after BCR-ABL inhibition, not necessarily dependent on mutagenesis per se.

### Combination of ATRA with tyrosine kinase inhibitors blocked CML acquired resistance through BCR-ABL mutation

**Figure 5:**
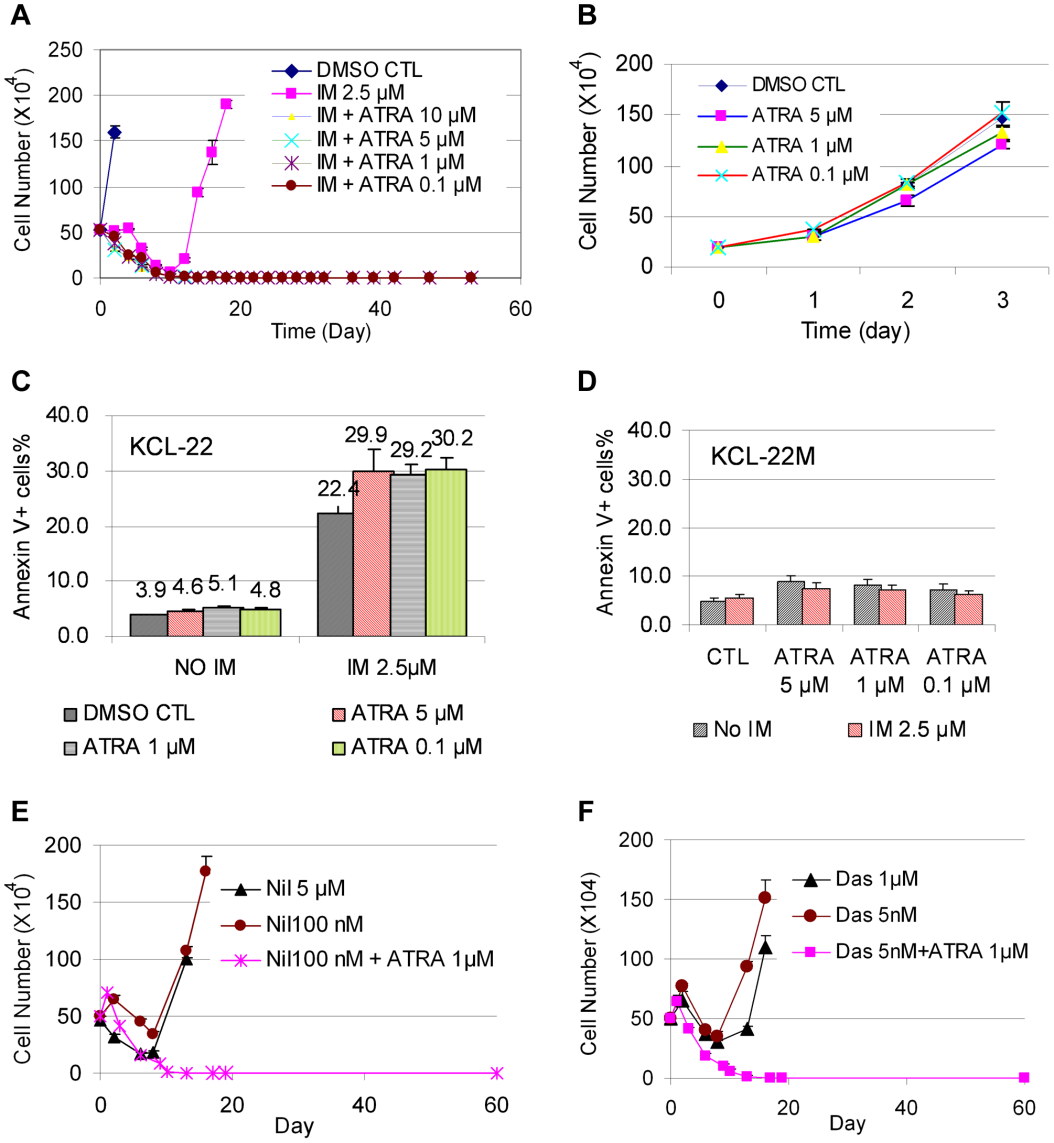
ATRA co-treatment blocked KCL-22 cell relapse on TKIs. (A) KCL-22 cells were treated with imatinib (IM) alone or in combination with ATRA at different concentrations, 0.1, 1, 5 and 10 µM. Only cells with IM treatment alone relapsed. (B) Growth curves of KCL-22 cells on treatment of ATRA. (C, D) Apoptosis of KCL-22 (C) and KCL-22M (D) cells treated with ATRA alone or in combination with IM. (E, F) KCL-22 cells were treated with nilotinib (E) or dasatinib (F) alone or in combination with ATRA. Cells for nilotinib or dasatinib treatment alone relapsed.

KCL-22 cells are immature myeloid progenitor cells [27]. We set to explore if forced differentiation may affect CML acquired resistance by testing the effect of ATRA that can induce partial myeloid differentiation. ATRA is a therapeutically effective drug in treating APL patients by inducing differentiation in promyelocytic leukemia-retinoic acid receptor (PML-RAR) positive APL cells in which the differentiation is blocked [15]. We first examined whether ATRA co-treatment would prevent mutation acquisition and CML cell relapse on imatinib. We treated KCL-22 cells with 2.5 µM imatinib in combination with various concentrations of ATRA. ATRA co-treatment at concentrations of 0.1∼10 µM effectively blocked KCL-22 relapse (Figure 5A). ATRA alone, at 5 µM and lower concentrations, did not significantly inhibit the growth or induce apoptosis of KCL-22 cells (Figure 5B, C) and did not change cell cycle (not shown). ATRA co-treatment slightly increased the apoptosis rate upon imatinib treatment in KCL-22 cells (Figure 5C), but did not significantly affect apoptosis in KCL-22M cells (Figure 5D). In addition, KCL-22 cells relapse on the treatment with second generation tyrosine kinase inhibitors nilotinib and dasatinib through acquisition of T315I mutation [12]. We found that 1 µM ATRA co-treatment with nilotinib or dasatinib effectively blocked KCL-22 cell relapse as well (Figure 5E, F).

**Figure 6:**
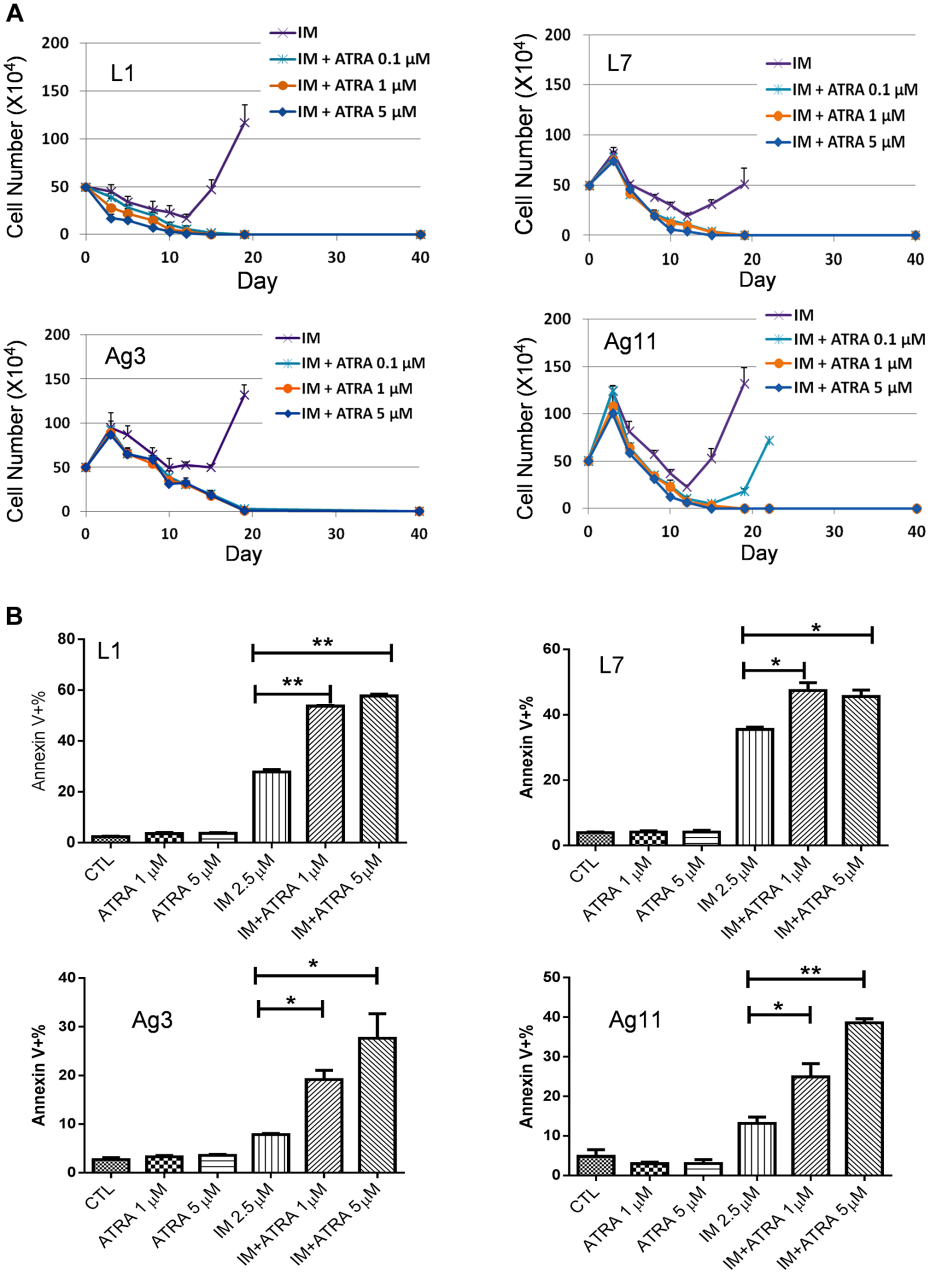
Effects ATRA treatment on CML cells acquiring different BCR-ABL mutations or no mutations. (A) Effects of ATRA on cell relapse on 2.5 µM IM in L1, L7, Ag3 and Ag11 cell lines. (B) Effects of ATRA in combination with IM on apoptosis induction of L1, L7, Ag3 and Ag11 cells.

To determine if ATRA co-treatment may work for inhibiting CML acquired resistance by other BCR-ABL mutations or non-mutation-mediated resistance, we treated four CML resistance cell lines that were originated from KCL-22 cells but developed resistance differently: L1, L7 and Ag11 lines through E255K, Y253H and T315I BCR-ABL mutations respectively, and Ag3 line through non-BCR-ABL mutation mediated mechanism [11]. We found that ATRA at 1 µM and higher concentrations blocked relapse of all four cell lines on IM (Figure 6A). Interestingly, L1, L7, Ag3 and Ag 11 cells were more susceptible for apoptosis induction than KCL-22 cells when ATRA was combined with IM (Figure 6B). However, apoptosis induction was not enhanced by ATRA/IM combination in K562 and KU812 CML cell lines (data not shown). Together, these results indicate that combination of ATRA with a tyrosine kinase inhibitor can prevent acquired resistance through BCR-ABL mutations, and may also inhibit resistance by certain non-mutation-mediated mechanisms.

### Rapid development of CML acquired resistance through BCR-ABL mutation

**Figure 7:**
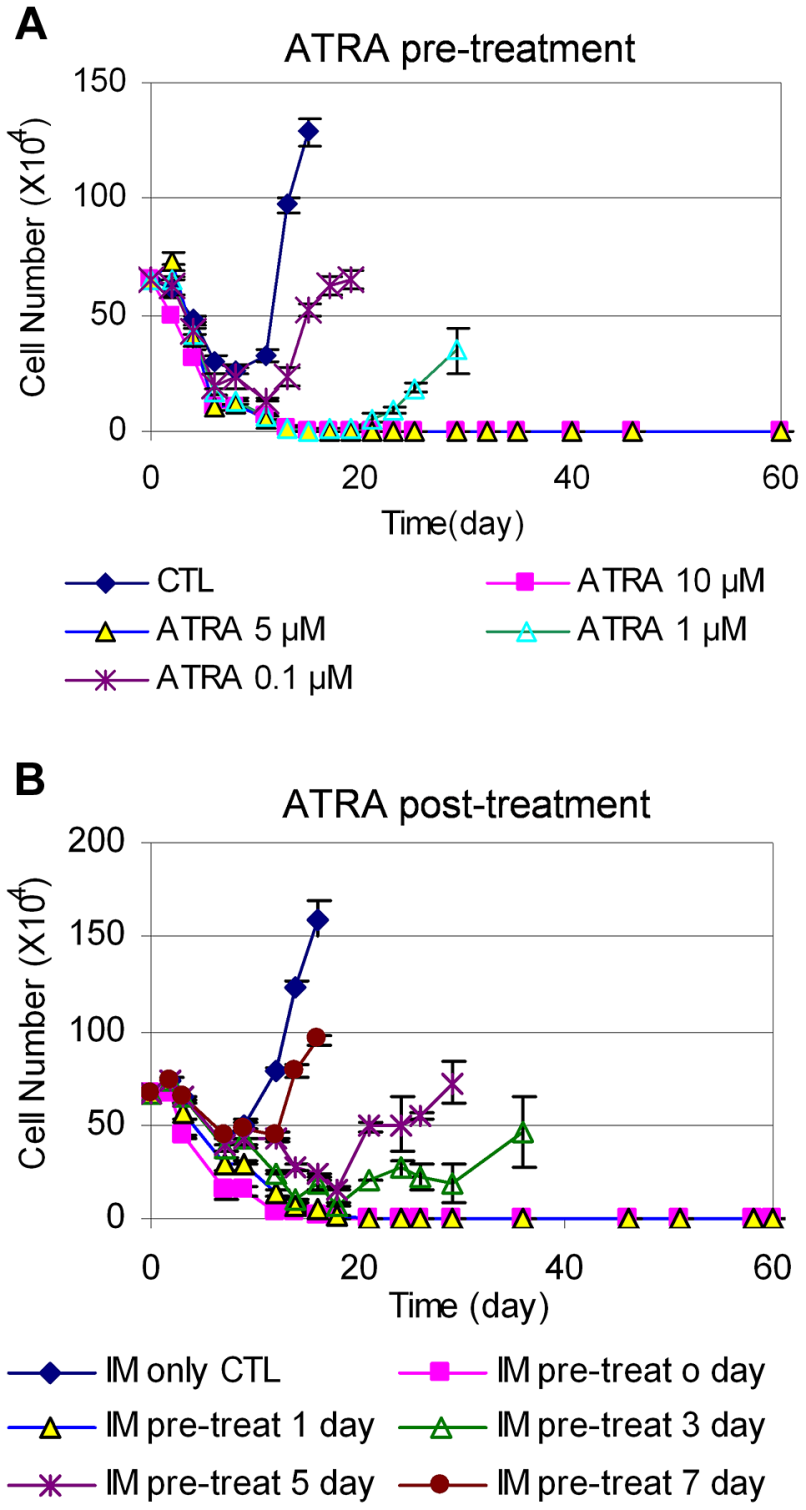
Timing of ATRA treatment and acquisition of BCR-ABL mutation. (A) KCL-22 cells were treated with DMSO (CTL) and 0.1, 1, 5 or 10 µM ATRA for 24 h. Cells were harvested and washed thoroughly with phosphate-buffered saline, and then replated with 2.5 µM IM without ATRA for relapse assay. (B) After initiation of KCL-22 cell treatment with 2.5 µM IM for 0, 1, 3, 5 and 7 days, 1 µM ATRA was added. Cell count was performed from the initiation of IM treatment.

We next examined how the timing of ATRA treatment may affect CML cell relapse. We treated KCL-22 cells with 0.1, 1, 5, and 10 µM ATRA for 24 h, 48 h or 72 h, followed by removing the drug and washing cells thoroughly. The ATRA-primed cells were then treated with 2.5 µM IM in the absence of ATRA. As shown in Figure 7A, 24 h pre-treatment with 5 or 10 µM ATRA completely blocked cell relapse whereas 1 µM ATRA significantly delayed the relapse. The same results were seen for ATRA pre-treatment for 48 and 72 h (Figure S4). These data suggest that transient (24 h) exposure to ATRA can significantly alter the ability of KCL-22 cells to acquire BCR-ABL mutations for IM resistance, even though the effect is less pronounced with lower concentrations of ATRA than when ATRA and IM are used simultaneously.

We then determined the effect of adding ATRA after the initiation of IM treatment. KCL-22 cells were treated with 2.5 µM IM first, followed by the addition of 1 µM ATRA at different time points. Interestingly, ATRA was fully effective only if it was added simultaneously with imatinib or one day after the start of imatinib, and ATRA addition at 3 days after the start of imatinib or later could not block the relapse (Figure 7B). These results suggest that *de novo* BCR-ABL mutation acquisition occurs rapidly, and may be mostly completed within 3 days of imatinib treatment; once completed, addition of ATRA can not block cell relapse.

### ATRA-induced CD38 expression changed NAD metabolism, SIRT1 function and CML acquired resistance

**Figure 8:**
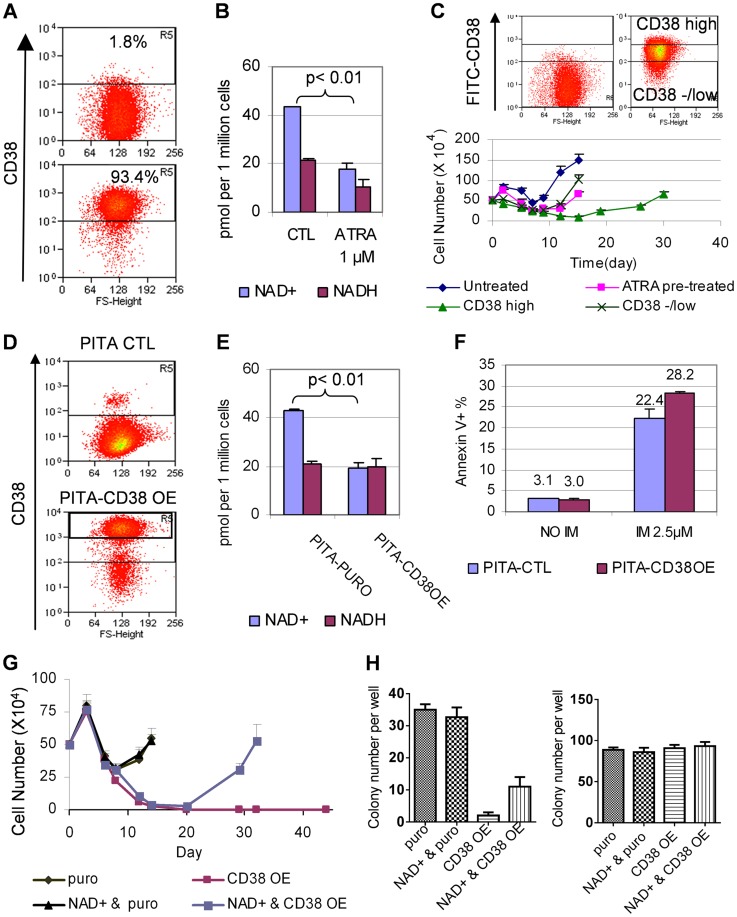
CD38 expression affected cellular NAD^+^ levels and KCL-22 cell relapse. (A) A representative flow cytometry profile showed dramatic increase of CD38 expression in KCL-22 cells in response to 1 µM ATRA for 24 h. (B) NAD^+^ levels in KCL-22 cells treated with 1 µM ATRA for 24 h. (C) CD38 levels affected CML cell relapse. Top: KCL-22 cells were treated with 1 µM ATRA for 24 h, and cells were sorted for high vs low/- CD38 expression. Bottom: The sorted cells were used for relapse assay with 2.5 µM IM. For comparison, KCL-22 cells without ATRA treatment (untreated) and ATRA pretreated but not sorted cells (ATRA pretreated) were also analyzed simultaneously. (D) Ectopic CD38 expression in KCL-22 cells. Cells were transduced with a PITA lentiviral vector for CD38 over-expression (CD38 OE) or the empty vector PITA-puro as control (CTL). Transduced cells were enriched by puromycin selection and analyzed by flow cytometry. (E) KCL-22 cells with high ectopic CD38 expression were sorted from the gate R5 of the lower panel in (D), and used for analysis of NAD^+^. The unsorted, empty PITA-puro vector transduced cells were used as control. (F) Apoptosis analysis of high ectopic CD38 expressing cells vs control as described in (E) with and without IM treatment. (G) Relapse assay with 2.5 µM IM for high ectopic CD38 expressing cells (CD38 OE) vs control (puro). For NAD^+^ rescue, 50 µM NAD^+^ was supplied at the beginning with IM. (H) Soft agar colony formation assay. Left: 1 million cells per well were seeded with 2.5 µM IM in the presence or absence of 50 µM NAD^+^. Right: 500 cells per well were seeded without IM in the presence or absence of 50 µM NAD^+^.

ATRA can induce dramatically increased expression of CD38, a leukocyte differentiation antigen and cell marker for committed hematopoietic progenitor cells [28], [29]. Indeed, we found that ATRA at concentrations as low as 0.1 µM efficiently converted KCL-22 cells from CD38^−^ to CD38^+^ (Figure 8A and Figure S5), consistent with its role in promoting myeloid differentiation. Noticeably, early studies suggested that ATRA can stimulate myeloid differentiation of blast crisis and chronic phase CML cells *in vitro* and *in vivo* [30], [31]. Besides being a cell marker, CD38 is also a potent cellular NAD^+^ regulator that hydrolyzes NAD^+^ to make cyclic ADP ribose [17]. Knockout of CD38 in mice significantly increases intracellular NAD^+^ content and SIRT1 activity [32], [33]. Given that SIRT1 plays a crucial role in promoting acquisition of BCR-ABL mutation [12], we hypothesized that ATRA-induced CD38 expression may impact cellular NAD^+^ metabolism and SIRT1 functions, and thus influence BCR-ABL mutation acquisition.

Consistent with increased CD38 expression, we found that cellular NAD^+^ levels were significantly reduced upon ATRA treatment (Figure 8B). We further sorted CD38^high^ and CD38^low/negative^ populations from KCL-22 cells after 24 h-treatment with 1 µM ATRA (Figure 8C). The sorted cells were then subjected to IM treatment in the absence of ATRA. CD38^high^ population showed a much more delayed relapse on IM treatment compared to CD38^low/negative^ population (Figure 8C), indicating that CD38 may play a role in CML cell relapse on imatinib.

To examine if CD38 may have direct contribution, we constructed a CD38 overexpression cassette in the PITA-puro lentiviral vector. KCL-22 cells were transduced with the CD38-expressing or control vector and enriched by puromycin selection (Figure 8D). CD38 over-expression significantly reduced cellular levels of NAD^+^, but not NADH (nicotinamide adenine dinucleotide plus hydrogen) (Figure 8E). CD38 over-expressing cells showed no difference in proliferation and survival compared to mock-transduced cells, but displayed slightly increased apoptosis on the IM treatment (Figure 8F and not shown), similar to that seen with ATRA treatment (Figure 5C). The puromycin enriched CD38 over-expressing cells exhibited a moderate delay in relapse on IM treatment and significant reduction of IM resistant soft agar colony formation (Figure S6). To remove interference from the remaining CD38^−^ cells in puromycin enriched cells, we flow-sorted CD38^+^ cells (gate R5 in the lower panel of Figure 8D). In the sorted CD38 over-expressing population, the cell relapse on IM was completely blocked in liquid culture, and this effect was partially reversed by supplying NAD^+^ to the culture medium (Figure 8G). Similarly, the IM resistant colony formation on soft agar was eliminated in the sorted CD38 over-expressing cells, and the effect was partially rescued by NAD^+^ supplement (Figure 8H). Our results suggest that CD38 expression in CML cells can inhibit the ability of the cells to acquire BCR-ABL mutations upon IM treatment through regulating NAD^+^ metabolism.

**Figure 9:**
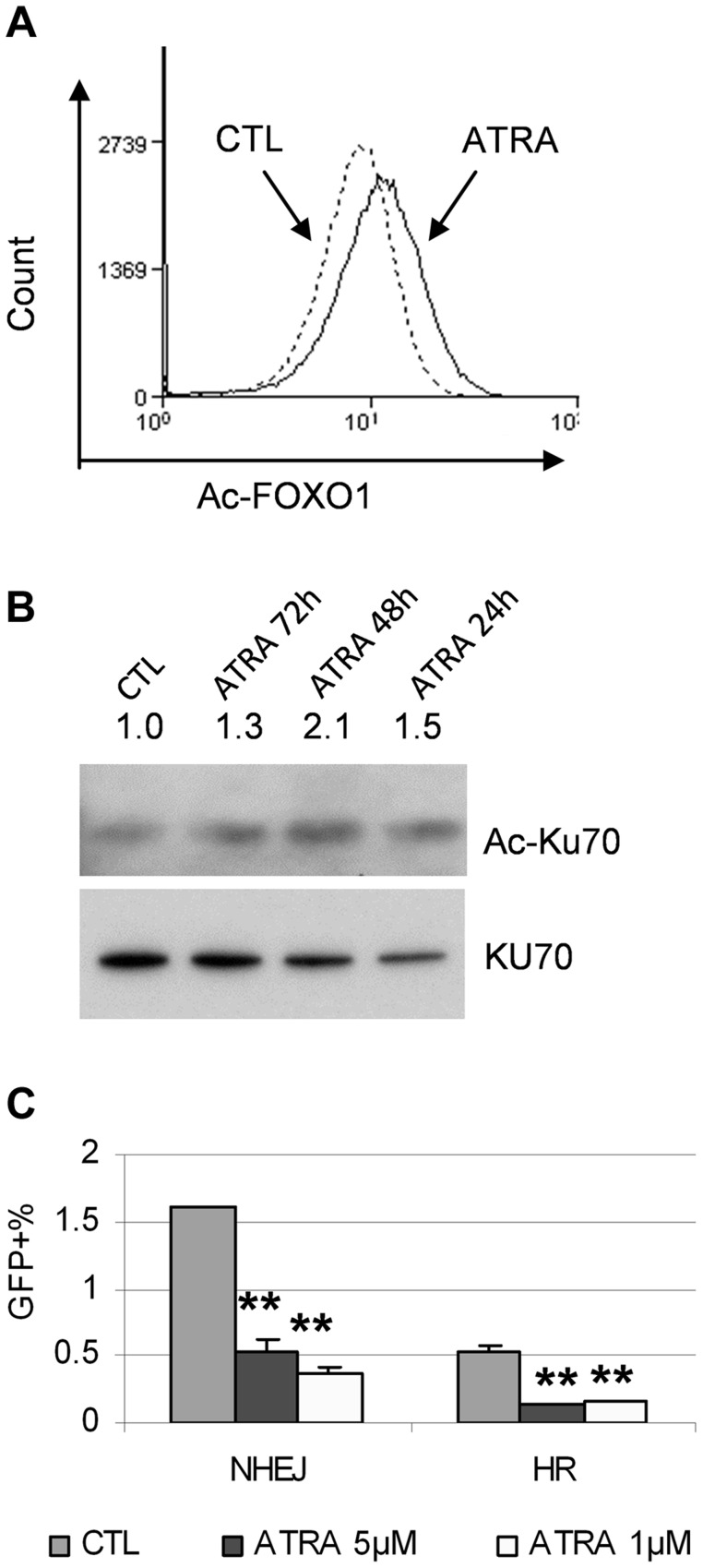
ATRA treatment affected SIRT1 functions. (A) FOXO1 acetylation change in response to 1 µM ATRA was analyzed by flow cytometry with an acetylated FOXO1 antibody. (B) Ku70 acetylation change in response to mock or 1 µM ATRA for 24 to 72 h was analyzed by immunoprecipitation with a Ku70 antibody and Western blot with a pan-acetyl lysine antibody. (C) NHEJ and HR reporter assays in KCL-22 cells in response to 1 µM ATRA treatment.

SIRT1 promotes aberrant DNA damage repair and facilitates BCR-ABL mutation acquisition [12]. However, we did not observe significant change of SIRT1 protein levels in KCL-22 cells upon ATRA treatment (not shown). To examine the impact of NAD^+^ reduction on SIRT1 enzymatic activity upon ATRA treatment, we analyzed acetylation of SIRT1 substrates FOXO1 and Ku70. As shown in Figure 9A and B, FOXO1 acetylation was increased with ATRA treatment by flow cytometric analysis and Ku70 acetylation was increased by immunoprecipitation and Western blotting, suggesting inhibition of SIRT1 activity. We previously developed DNA damage repair reporter assays for non-homologous end joining (NHEJ) and homologous recombination (HR) repair in KCL-22 cells, and showed that SIRT1 promotes both NHEJ and HR repair in these cells [12]. We found that ATRA treatment significantly reduced both NHEJ and HR repair in KCL-22 cells (Figure 9C), consistent with the reduction of SIRT1 activity. Altogether, our results indicate that ATRA-induced cellular differentiation blocks BCR-ABL mutation acquisition in CML cells, in which CD38 activation appears to play an important role by reducing cellular NAD^+^ content and thus inhibiting SIRT1 activity.

### CD38 expression in primary human CML progenitor cells

**Figure 10:**
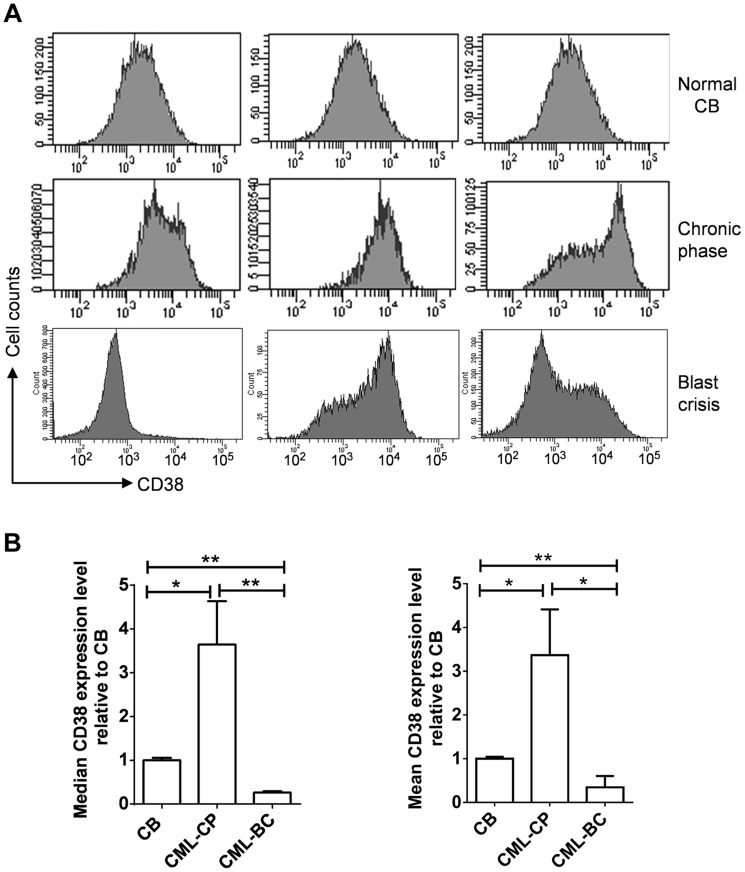
CD38 expression in primary CD34^+^ normal and CML progenitor cells. (A) Flow cytometry plots showing CD38 expression in CD34^+^ cells isolated from normal cord blood (CB), chronic phase (CP) CML and blast crisis (BC) CML. Three independent samples from each group were shown. (B) Comparison of mean and median CD38 levels in A after normalization to CB samples. Statistically significant difference was indicated by asterisks.

Given the roles of CD38 described above, we examined if CD38 expression may change during CML progression. CD38 was not in the Radich's 3415 “phase reporter” gene sets and its mRNA change was less significant [18]. We therefore analyzed CD38 protein expression from archived cell sorting data of CML and normal CD34^+^ progenitor cells that were collected on the same cell sorter with the same parameters after pre-enrichment by density gradient and immunomagnetic column separation [34]. Intriguingly, we found that overall CD38 protein levels were increased in chronic phase CML, but reduced in blast crisis CML (Figure 10). Besides, variation of CD38 expression among samples was larger in CML than in normal CD34^+^ cells. The increase of CD38 protein in chronic CML is reminiscent of previous findings that BCR-ABL enhances differentiation of long-term repopulating stem cells and loss of cell quiescence [35], [36], whereas reduction of CD38 protein in blast crisis CML is in line with the reduced myeloid differentiation described by Radich et al [18]. Reduction of CD38 in blast crisis cells may allow sufficient NAD^+^ supply for markedly increased SIRT1 expression in blast crisis CML [37].

## Discussion

Our microarray and exome sequencing analysis of the paired T315I BCR-ABL mutant KCL-22M cells vs parent KCL-22 cells not only provided data supporting our previous experimental observations, but also revealed several new features of the process of BCR-ABL mutation acquisition. 1) Mutation acquisition is accompanied by activation of several drug resistance pathways. 2) Mutant cells exhibit reduced expression of myeloid differentiation genes. 3) There are a number of exome-wide point mutations and small indels accumulated in the mutant cells, but these genetic lesions don't seem to determine the changes in global gene expression; instead, they may occur in parallel or separately. The gene expression changes suggest possibly a certain degree of epigenetic reprogramming in the parental KCL-22 cells in response to IM treatment. Intriguingly, drug resistance in a subset of non-small cell lung cancer patients is associated with epithelial-to-mesenchymal transition that transforms non-small cell lung cancer into small cell lung cancer [38], which also suggests possible epigenome reprogramming in that setting.

In line with potential epigenetic reprogramming during mutation acquisition, we showed that forced differentiation of KCL-22 cells by ATRA effectively blocked acquisition of resistance in KCL-22 cells on IM treatment. ATRA plays a wide variety of roles in regulating cell growth, development and differentiation. In the normal hematopoietic system, ATRA enhances the growth and differentiation of hematopoietic progenitors of the granulocytic lineage, and increases maintenance and self-renewal of murine long-term repopulating hematopoietic stem cells [39], [40]. ATRA induces terminal differentiation of abnormal promyelocytes in APL and is the first-line drug for treating APL patients [41]. ATRA also induces myeloid differentiation of hematopoietic stem/progenitor cells from blast crisis CML *in vitro* and *in vivo* [30], and from chronic CML *in vitro* [31]. As a pre-IM era drug, ATRA had only limited effect on CML treatment either in a single drug or combination with other drugs [42]–[44]. However, its effect with IM combination has not been tested. The potent effect of ATRA to prevent acquisition of BCR-ABL mutation shown in the present study suggests that ATRA could be a simple and effective agent to use in combination with tyrosine kinase inhibitors to prevent acquisition of resistance and improve outcomes of CML, particularly in the advanced phases. Although advanced CML without a history of IM treatment is extremely rare in developed countries nowadays, such patients are still more frequently seen in some developing countries and many of them have no or limited access to TKIs, especially newer generation drugs. A simple ATRA combination may significantly benefit these patients. For the clinical purpose, ATRA should be better administrated together with tyrosine kinase inhibitors. The concentrations of both IM and ATRA illustrated in this study are clinically achievable. The dosage of ATRA for CML patients ranges from 45 to 175 mg/m^2^/day [42]. Following 45 mg/m^2^/day oral dose of ATRA, the peak plasma concentration is from 0.1 to 8 µM, with a median peak concentration of about 1 µM [45]. Similarly, IM given to chronic phase CML patients at 400 mg/day produces an average peak plasma concentration of 4.4 µM and trough concentration of 2.0 µM [46], and they will increase further in advanced phase patients with higher IM doses.

Mechanistically, ATRA efficiently induced partial myeloid differentiation of KCL-22 cells with robust stimulation of CD38 expression. CD38 is widely expressed in most mature blood and immune cells and 90–99% of CD34^+^ hematopoietic progenitor cells [47], [48]. In myeloid cells, expression of CD38 can be efficiently modulated by ATRA, 1α,25-Dihydroxyvitamin D3, IFN-α/γ, rotenone, etc. [28], [49], [50]. Other than being a cell surface marker, CD38 is also recognized as a multifunctional enzyme that uses NAD^+^ as a substrate to generate second messenger and participates in signal transduction pathways involved in the regulation of cell growth and differentiation [17]. Most notably, it is the main cellular NADase in mammals and regulates cellular levels of NAD^+^ in multiple tissues and cells. High levels of CD38 expression depleted cellular NAD^+^ and suppressed BCR-ABL mutation acquisition in KCL-22 cells. Importantly, such effect can be partially rescued by supplying NAD^+^ directly to the culture medium. Therefore, for the first time, to our knowledge, we revealed the status of cellular differentiation and CD38 expression can change the ability of cancer cells to acquire resistant genetic mutations to a therapeutic agent.

NAD^+^ is required for functions of many cellular enzymes including sirtuins. Sirtuins regulate multiple physiological processes such as cell proliferation, survival, DNA damage repair, energy metabolism, insulin secretion, aging and longevity [51], [52]. Sirtuins have diverse and often opposing roles in human cancer [53], [54]. SIRT1 is the most studied sirtuin protein. SIRT1 is up-regulated in malignant cells in a variety of cancers [53], [54], and has a particularly important role in cancer drug resistance [55]. We have shown that BCR-ABL transformation activates SIRT1 through both kinase dependent and independent manners [37]. SIRT1 inhibition suppresses CML progression and sensitizes CML stem cells to IM–induced apoptosis [37], [56]. SIRT1 also promotes acquisition of BCR-ABL mutation for IM resistance in CML cells through facilitating error prone DNA damage repair [12]. Consistently, our current study showed that reduction of NAD^+^ lowered SIRT1 enzymatic activity and DNA damage repair in KCL-22 cells, which may lead to inhibition of KCL-22 cell relapse on IM. Important to note that SIRT1 is a key epigenome regulator and deacetylates multiple histone substrates [53]. In this regard, however, it is unclear how and if SIRT1 may play a role in epigenome reprogramming of KCL-22 cells, which remains an interesting subject for future investigation. In addition, high levels of CD38 expression was required to block KCL-22 cell relapse and NAD^+^ supplementation only partially rescued the phenotypes in CD38 over-expressing cells, suggesting that additional effects of ATRA-induced differentiation or CD38 expression may have on these cells.

CD38 is an important cell marker in human hematopoietic stem cells (HSCs) [48]. CD34^+^CD38^−^ bone marrow cells are highly enriched for long-term repopulating HSCs while CD34^+^CD38^+^ cells are more committed progenitor cells. These same markers also apply to CML leukemic stem cells. Given that CD38^−^ cells may have higher NAD^+^ levels and more active SIRT1, CD34^+^CD38^−^ CML stem cells might have more permissive cellular environment for acquisition of genetic mutations. This view is in line with a recent finding that primitive leukemic stem cells may be the origin of the cells acquiring resistant mutations in chronic CML [57]. In stark contrast, the majority of chronic CML patients benefit from years' IM treatment without acquiring BCR-ABL mutation even though CML stem cells are persistent. Although the precise mechanisms underlying this discrepancy are not known, there may be several explanations. First, SIRT1 is activated much more in advanced and blast crisis than in chronic phase CML CD34^+^ cells [37]. The lower levers of SIRT1 activation in chronic CML may be insufficient to promote strong mutagenic response. Second, CD38 levels are elevated in chronic CML, reducing NAD^+^ supply as mentioned above. Third, acquisition of BCR-ABL mutation may ironically inhibit its transformation ability in CD34^+^CD38^−^ cells in the chronic phase CML, somewhat similar to that in KCL-22M cells described above. Fourth, chronic CML stem cells may have distinct epigenomic composition of the translocation locus unfavorable for mutagenic response to the treatment. In spite of these, a small percentage of chronic CML patients may eventually relapse with acquisition of BCR-ABL mutations. It remains possible that ATRA/TKI combination could also benefit the management of the disease in chronic phase, considering 1) relapsed chronic CML patients with or without acquisition of BCR-ABL mutations assume gene expression patterns closely related to blast crisis CML and likely with reduced differentiation [18]; 2) ATRA can still induce myeloid differentiation of chronic CML stem/progenitor cells [31] even though these cells have elevated CD38 levels as described above; 3) ATRA/TKI combination may inhibit certain acquired resistance in the absence of BCR-ABL mutations as described above. However, as in blast crisis CML, the maximal benefit of ATRA would be when it is applied with TKIs from the beginning or before mutations are acquired.

Besides CML, CD38 expression is used as a prognostic indicator in chronic lymphocytic leukemia (CLL), and CD38 expression is closely related with *Ig V* gene hypermutation status [58]. Patients with lower percentage of CD38^+^ B-CLL cells carry higher *Ig V_H_* mutations. It is believed that down-regulation of CD38 may promote this hypermutation phenotype, but its precise role remains elusive [59]. It would be interesting to determine if SIRT1 may be involved in such regulation.

Finally, our study with ATRA on KCL-22 cell relapse revealed that acquisition of BCR-ABL mutation is a rapid process, likely completed within three days of IM treatment. Although it has been proposed that preexisting mutations account for acquired resistance in primary human cancers by mathematical model studies [60], [61], our finding of rapid mutation acquisition calls for caution of such a notion because the variation in timing of relapse in CML and other cancer patients in several weeks [60], [61] would prevent distinction of pre-existing mutations from *de novo* acquired mutations. The precise mechanisms for rapid mutagenesis and critical timing of ATRA remain to be further determined. We previously showed that early apoptotic cells induced by IM sustain the highest levels of reactive oxygen species [11]. We hypothesized that a small fraction of early apoptotic cells may abolish apoptotic program while suffering substantial DNA damage, leading to mutagenesis [14]. The present study provides evidence of a large number of genome-wide genetic lesions likely as a consequence of massive DNA damage, and that upregulation of multiple resistant gene expression pathways perhaps by rapid epigenome reprogramming may help these cells abandon apoptotic program. ATRA treatment perhaps not only promotes myeloid differentiation but also provides a counter epigenetic reprogramming for global gene expression, which occurs in conjunction with reducing DNA damage repair, and thus its timing is crucial. Further studies will be needed to uncover this mystery.

In summary, we have used microarray-based gene expression analysis to show that global gene expression changes accompany BCR-ABL mutation acquisition in response to IM treatment, and that forced differentiation of CML cells with ATRA stimulates CD38 expression and blocks BCR-ABL mutation acquisition and CML cell relapse on IM in part through down regulation of NAD^+^ levels and SIRT1 activity. This study has translational implication for potential use of ATRA in combination with BCR-ABL inhibitors to improve CML treatment.

## Materials and Methods

### Cell lines and drugs

CML cell lines KCL-22, K562 and KU812 were purchased from German Collection of Cell Cultures, Braunschweig, Germany, and grown in RPMI 1640 medium with 10% fetal bovine serum (Hyclone, SH30071.03). KCL-22M cells harboring T315I BCR-ABL mutation were derived from KCL-22 cells [11]. Imatinib, nilotinib and dasatinib were purchased from LC Laboratories (Woburn, MA). ATRA was purchased from Sigma.

### Imatinib resistance assay

Imatinib resistance assay was performed as described before [11]. In brief, one half million KCL-22 cells were seeded in 1 ml medium per well in 24-well plates, and treated with IM alone or in combination with other drugs. Cell viability was assessed by trypan blue exclusion and cell count was performed every 3–5 days. Mutant cell clonogenic assay was performed using a standard two-layer soft agar culture (0.6% agarose for the bottom layer and 0.35% agarose for the top layer) by seeding one million cells per well with imatinib in six-well plates. For plating efficiency control, 500 cells per well were seeded. Colonies were scored after staining with 0.005% Crystal Violet.

### Microarray gene expression analysis

Total RNA was extracted from KCL-22 and KCL-22M cells using Trizol (Invitrogen). Triplicate RNA samples each were submitted to the City of Hope Functional Genomic Core for microarray expression analysis. Quality of RNA samples was checked using Agilent Bioanalyzer 2100. Samples were processed with GeneChip Two-Cycle Target Labeling and Control Reagents (Affymetrix, Santa Clara, CA), and hybridized to Affymetrix GeneChip Human Gene 1.0 ST Arrays. Microarray data analysis was performed using Partek Genomics Suite 6.6 (Partek, Inc.). RMA algorithm was adopted to normalize and summarize the intensities of probes into gene-level expression. Appropriate statistical model was used to identify differentially expressed genes between KMP3 and KP7 sample groups. Genes with significantly differential expressions were selected by use of a cutoff of a 1.5-fold change in the level of expression and p-value cutoff of <0.05.

The normalized signals for the whole array for the 6 samples were analyzed using Gene Set Enrichment Analysis (GSEA) software to determine the enriched pathways between KMP3 and KP7. Given the limit of sample size, gene-set permutation was adopted with 1000 permutations against molecular signatures database v3.0. The tested signatures database included C1 positional gene sets, C2CP canonical pathways, C2CGP chemical and genetic perturbations and C5 GO gene sets. The genes showing altered expression were categorized and further investigated by enrichment analysis on the basis of their cellular components, biological processes, molecular functions, and canonical pathways using the Ingenuity Pathways Analysis (Ingenuity, Mountain View, CA) software. To obtain comprehensive view for the differentially expressed genes, core analysis was performed including network generation, functional analysis and canonical pathway analysis on the filtered (|FC|>1.5, P<0.05) genes. Microarray study was carried out in City of Hope Integrative Genomics Core.

### Real-time qPCR analysis

To validate the array data, SYBR green real-time qPCR was performed. Total RNA was extracted using Trizol (Invitrogen) and residual genomic DNA was removed by DNase-I treatment. One microgram of DNase-I treated total RNA per sample was used for cDNA synthesis using SuperScript III First-Strand Synthesis System (Invitrogen) with oligo(dT). The synthesized cDNA was treated with RNase H for 20 min at 37°C to remove RNA. qPCR was performed in separate tubes using universal SYBR green master mixture (KAPA Biosystems) and gene specific primers on Applied Biosystems 2720 PCR thermal cycler. The PCR primers are listed in Supporting Table S5.

### Exome capture sequencing by Illumina Genome Analyzer (Solexa)

Three micrograms of genomic DNA was sheared by sonication using a Bioruptor (Diagenode). The resultant 150- to 250-bp fragmented DNA was end-repaired and ligated to Illumina adaptor oligonucleotides. Ligation products were purified and successfully ligated fragments were amplified with a 10-cycle of PCR. The enriched PCR products (500 ng) were subject to the exome capture procedure using the SureSelect Human All Exon v4 Target Enrichment Kit (solution magnetic bead capture) according to the manufacturer's protocols (Agilent Technologies, Inc.). Post-capture LM-PCR amplification was performed using the Herculase II Fusion DNA Polymerase (Agilent) for 10 cycles of amplification with primer set (Forward primer: 5′-CAAGCAGAAGACGGCATACG-3′; reverse primer: 5′-AATGATACGGCGACCACCGA-3′). After the final Agencourt Ampure XP bead (Beckman Coulter) purification, quantity and size of the library was analyzed using the Agilent Bioanalyzer 2100 DNA high Sensitivity chip. Library templates were prepared for sequencing using Illumina's cBot cluster generation system with TruSeq PE Cluster V3 Kit. Sequencing runs were performed in paired-end mode using the Illumina HiSeq 2000 platforms and TruSeq SBS V3 Kits. Image analysis and base calling were performed using Illumina's default pipeline. Sequencing runs generated approximately 130 million paired reads for each sample.

### Illumina data analysis

The sequences were aligned to the human genome reference sequence hg19 using Novoalign. The sequences that were PCR duplicates (aligned to the same genome location) were removed by Picard. SNVs (single nucleotide variants) in each sample were identified and their concordance to dbSNP134 was assessed. KCL-22M specific SNVs were identified by comparing to the KCL-22 sample and then annotated by SeattleSeP annotation web site. The missense/nonsense/splice site SNVs were kept. This produced 3260 KCL-22M specific mutations and among them, 208 were coding exon mutations. For copy number analysis, the average coverage in each 10 k non-overlapping window was calculated for each sample and then CNVs (copy number variants) were identified by using DNAcopy's CBS algorithm. For detection of indels, Samtools pileup file was parsed to select the positions with indels. These positions were filtered to select candidate indels in KCL-22M with the following criteria: minimum coverage ≥10, indel coverage ≥3, and indel frequency ≥15%. The indels present at the same position in KCL-22 were removed. The candidate indels that fell into coding exons were selected. The indels were annotated at SeattleSeq annotation web site and the ones causing frame shift were kept (33 indels). To search for additional deletions, insertions, inversions, tandem duplications and other structural variants, we used Pindel [62]. To prevent possibility of false positives, indels reported by Samtools in KCL-22 cells were removed, and indels located at genomic loci without any reads in KCL-22 cells, which does not have enough information to conclude a specific indel in KCL-22M, were removed. Additional criteria included total coverage in KCL-22M ≥5 and indel frequency ≥20%. These resulted in 1643 deletion and 563 insertion that were KCL-22M specific. Pindel did not report any KCL-22M specific inversion, tandem repeats or large insertion that seemed to be real when visually inspecting the region or manually realign the reads to hg19 with Blat. The above identified mutations and indels have not been validated by targeted re-sequencing except for BCR-ABL mutation. Exome sequencing study was carried out in the City of Hope Integrative Genomics Core.

### Flow cytometry and protein analysis

For fluorescence-activated cell sorting or analysis, half million cells were stained with fluorophore conjugated antibodies in a buffer (0.5% bovine serum albumin in phosphate-buffered saline) for 15 min on ice. Antibodies used for analysis and sorting were: APC-anti CD38, FITC-anti CD38, PE-anti CD90, APC-anti-CD11b (BD Pharmingen), and rabbit polyclonal anti-acetylated FOXO1 (Santa Cruz Biotech). Apoptosis was analyzed by annexin-V (BD Pharmingen) staining. Flow cytometry was performed at the City of Hope Flow Cytometry Core.

For Western blot, rabbit monoclonal anti-human SIRT1 (Epitomics), mouse monoclonal anti-Ku70 (Neomarker) were used. To analyze Ku70 acetylation, we pulled down Ku70 from total cell lysate with anti-Ku70 and protein A/G plus-agarose beads (Santa Cruz) followed by acetylation detection with the rabbit anti-acetyl lysine antibody (Cell Signaling).

### Ectopic CD38 expression

CD38 cDNA was kindly gifted by Prof. Elena Zocchi and subcloned into PITA-puro lentiviral vector that contains a puromycin selection cassette. PITA-puro vector was used as a mock control plasmid. The lentiviral packaging for PITA-puro and PITA-CD38 was performed as described previously [11]. Transduction was typically carried out with multiplicity of infection around 5. To enrich the transduced cells, cells were treated with 2 µg/mL puromycin for 4 days and allowed to recover in normal medium for 6 days before analysis.

### DNA damage repair assay

The doxycycline inducible DNA damage repair report systems in KCL-22 cells with DR-GFP for HR repair and EJ5-GFP for NHEJ repair were established previously [12]. These cells were then transfected with PITA-SCR or PITA-CD38 lenti-virus for 16 h followed by addition of 5 ng/mL doxycycline to induce I-SceI expression. After another 48 h culture, the cells were analyzed by flow cytometry for GFP expression to determine the repair efficiency. GFP^+^ cells were the successfully repaired cells. To determine the effect of ATRA treatment on DNA repair, cells were incubated with ATRA for 3 days then co-treated with 5 ng/mL doxycycline for another 48 h before flow cytometry analysis.

## Supporting Information

Figure S1 Microarray data visualization and analysis plots. (A) Principal component analysis of the microarrays. Orange dots of KMP3 were for samples of KCL-22M cells and blue dots of KP7 for KCL-22 cells. (B) Volcano plot of t-test between KMP3 vs KP7. Probe sets with fold change >1.5 and p-value<0.05 were identified as significant and indicated by red color (significant: 1), and non-significant probe sets colored by blue (significant: 0).
(TIF)

Figure S2 GSEA enrichment plots of cell cycle related genes in CML progenitor cells.
(TIF)

Figure S3 IPA pathway analysis. (A) Top ten altered functional pathways identified by ingenuity pathway analysis (B) qPCR validation of gene expression affecting hematopoietic progenitors.
(TIF)

Figure S4 Effect of ATRA pre-treatment. KCL-22 cells were pretreated by ATRA for 48 and 72 h, and then analyzed in the absence of ATRA for relapse.
(TIF)

Figure S5 CD38 expression after ATRA treatment. Flow cytometry analysis of CD38 expression in KCL-22 cells treated with ATRA at concentrations indicated.
(TIF)

Figure S6 Effect of CD38 expression. Puromycin enriched, CD38 transduced KCL-22 cells (but not sorted) were analyzed for relapse on IM in liquid culture (A) and IM resistant soft agar colony formation (B). In B, left panel was plating efficiency with 500 cells/well in the absence of IM; right panel with 1 million cells/well in 2.5 µM IM.
(TIF)

Table S1 List of significant microarray probe sets.
(XLS)

Table S2 C1 positional gene set analysis.
(TIF)

Table S3 Coding exon mutations specific in KCL-22M cells.
(XLS)

Table S4 Coding exon indels specific in KCL-22M cells.
(XLS)

Table S5 qPCR primer sequences.
(TIF)
