# Correction: ATRA-Induced Cellular Differentiation and CD38 Expression Inhibits Acquisition of BCR-ABL Mutations for CML Acquired Resistance

**DOI:** 10.1371/journal.pgen.1005033

**Published:** 2015-03-05

**Authors:** The PLOS Genetics Staff

There is an error in Fig. 4 in which chromosome Y was incorrectly included in Fig. 4A,B of the female cells. This was caused by misalignment of a small number of Y-chromosome sequence. The corrected figure panels are attached here. This error changes genome-wide point mutation number from 3260 to 3093, and indel number from 2206 to 2204 in the main text; however, it does not affect the mutations or indels on coding exons and interpretation of the results or conclusions of the paper. The authors apologize for any confusion this may have caused.

**Fig 4:**
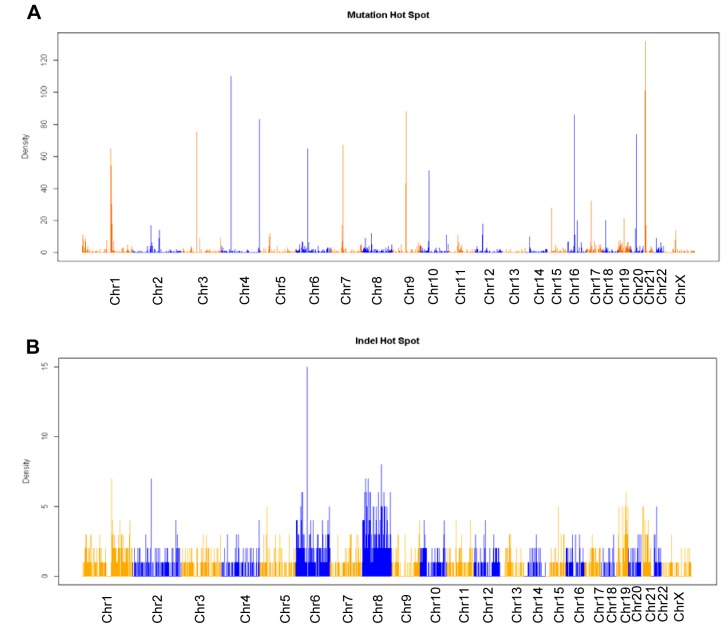
Exome sequencing analysis of KCL-22M vs KCL-22 cells. (A, B) Chromosomal distribution of point mutation (A) and indel (B) hot spots identified specifically in KCL-22M cells. The counts of point mutation or indel events in 1 Mb windows along each chromosome were drawn. (C) Venn diagram analysis of significant gene expression probe sets from Fig. 1A, point mutations and indels in coding exons. The numbers included 160 annotated probes from the 245 expression probe sets, 194 unique genes for a total of 208 point mutations in coding exons, and 56 accession numbers for a total of 33 unique genes bearing frame-shift indels in coding exons. None of 7 overlapping genes were on chromosome 4.
