# Supplementary figures and images for: ATRA-Induced Cellular Differentiation and CD38 Expression Inhibits Acquisition of BCR-ABL Mutations for CML Acquired Resistance
Source: PLoS Genet. 2014 Jun 26;10(6):e1004414. doi: 10.1371/journal.pgen.1004414 (PMC4072521; doi:10.1371/journal.pgen.1004414)

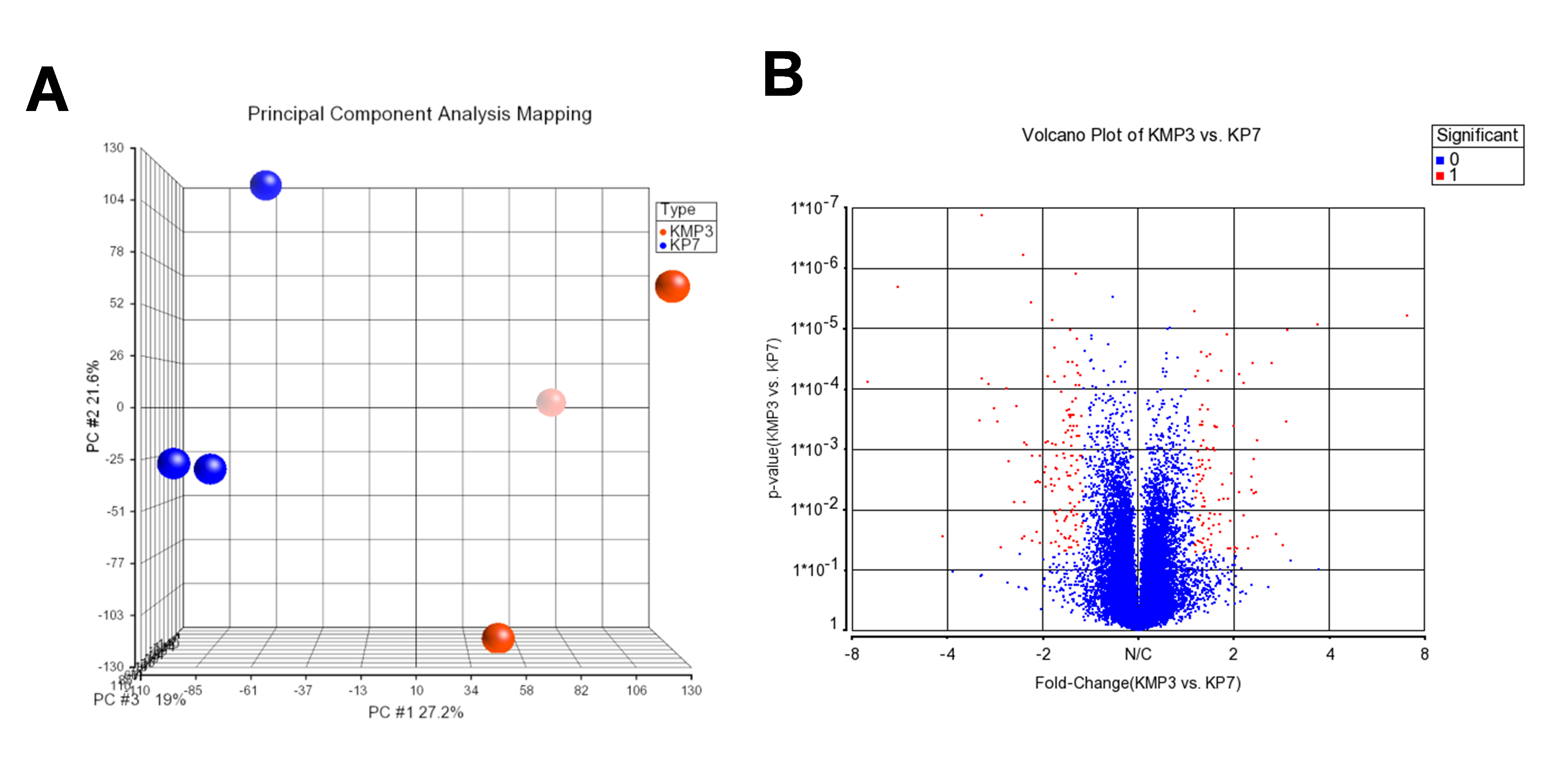

Supplement: Figure S1 — Microarray data visualization and analysis plots. (A) Principal component analysis of the microarrays. Orange dots of KMP3 were for samples of KCL-22M cells and blue dots of KP7 for KCL-22 cells. (B) Volcano plot of t-test between KMP3 vs KP7. Probe sets with fold change >1.5 and p-value<0.05 were identified as significant and indicated by red color (significant: 1), and non-significant probe sets colored by blue (significant: 0). (TIF) [file pgen.1004414.s001.tif]

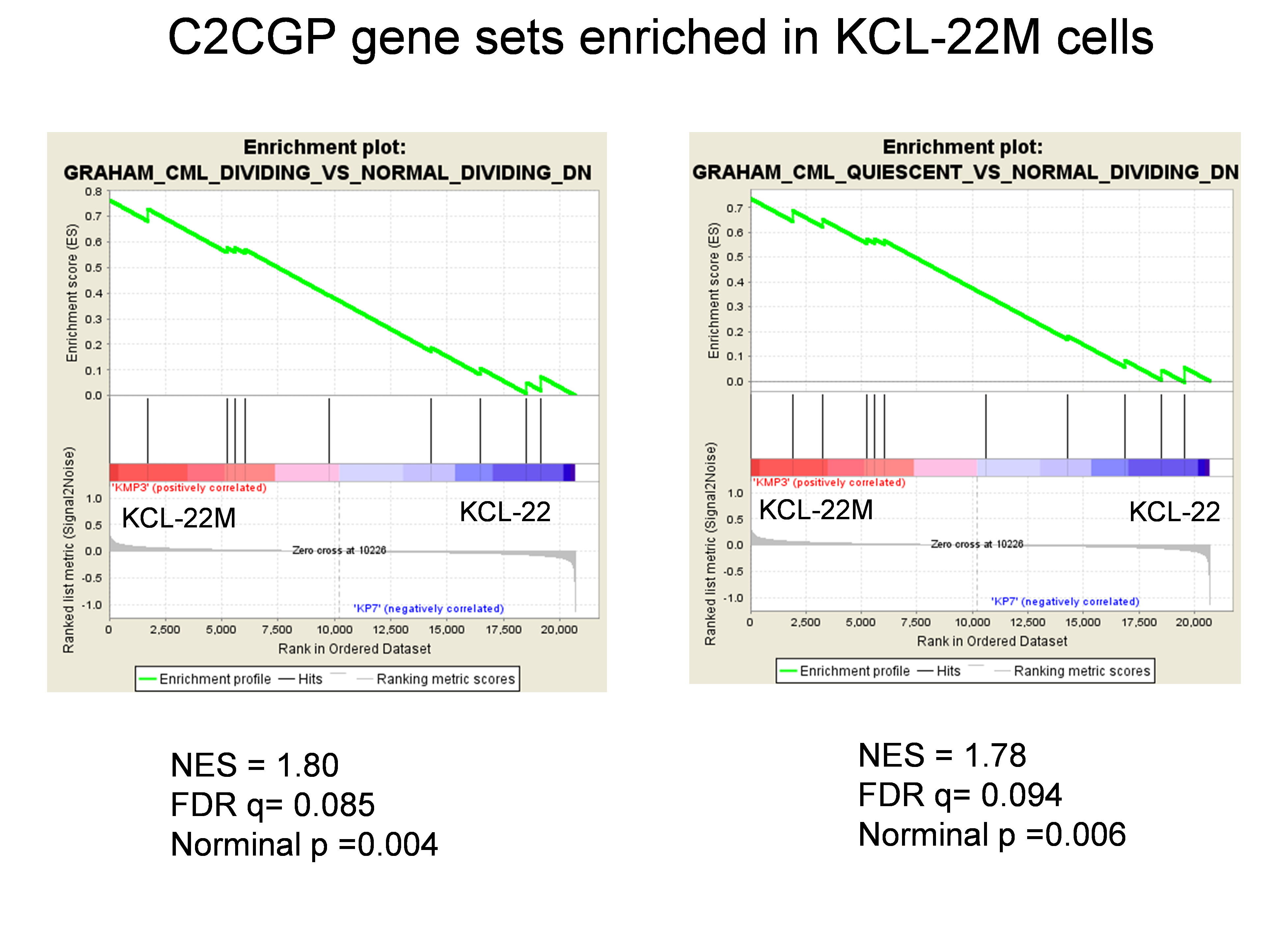

Supplement: Figure S2 — GSEA enrichment plots of cell cycle related genes in CML progenitor cells. (TIF) [file pgen.1004414.s002.tif]

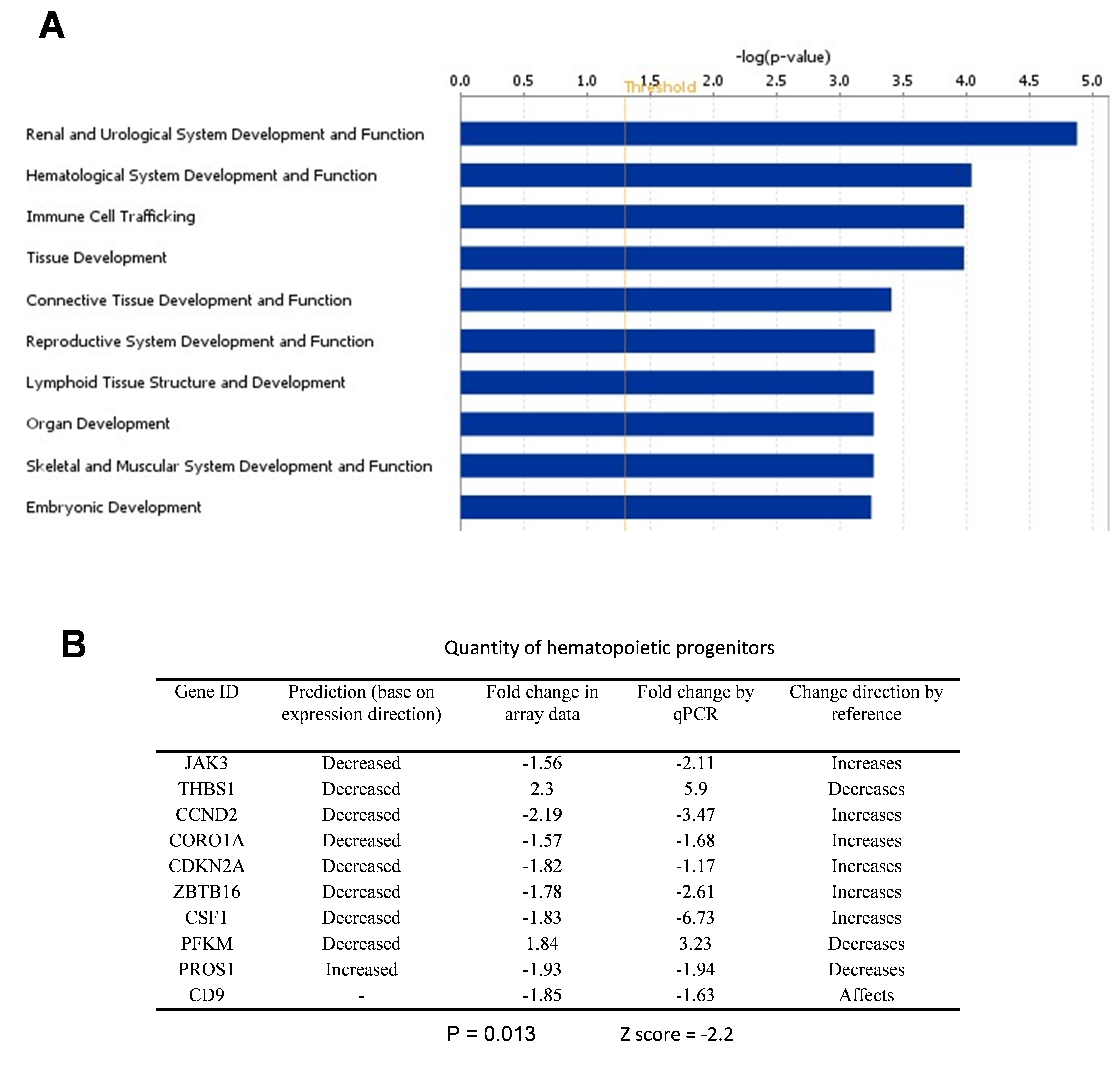

Supplement: Figure S3 — IPA pathway analysis. (A) Top ten altered functional pathways identified by ingenuity pathway analysis (B) qPCR validation of gene expression affecting hematopoietic progenitors. (TIF) [file pgen.1004414.s003.tif]

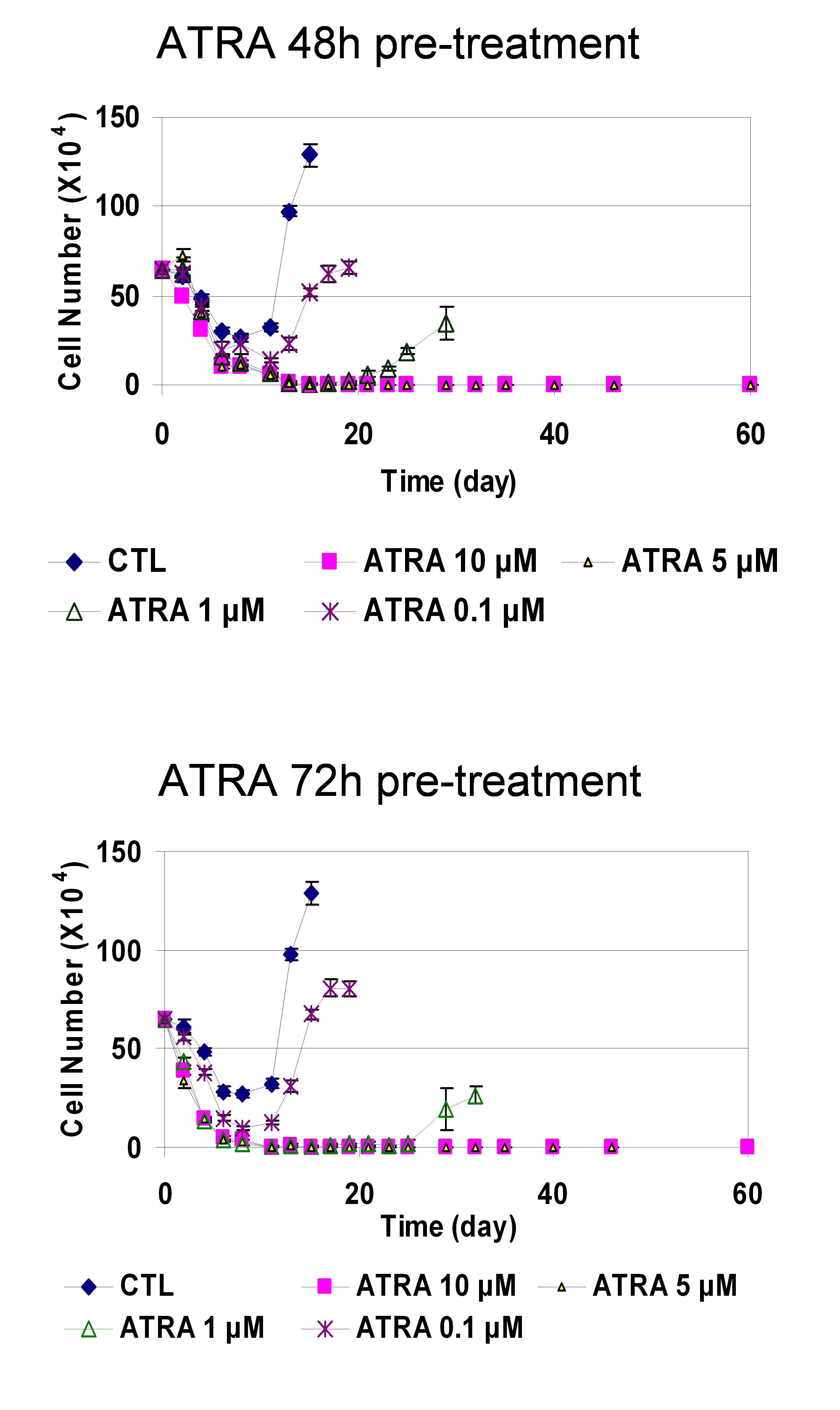

Supplement: Figure S4 — Effect of ATRA pre-treatment. KCL-22 cells were pretreated by ATRA for 48 and 72 h, and then analyzed in the absence of ATRA for relapse. (TIF) [file pgen.1004414.s004.tif]

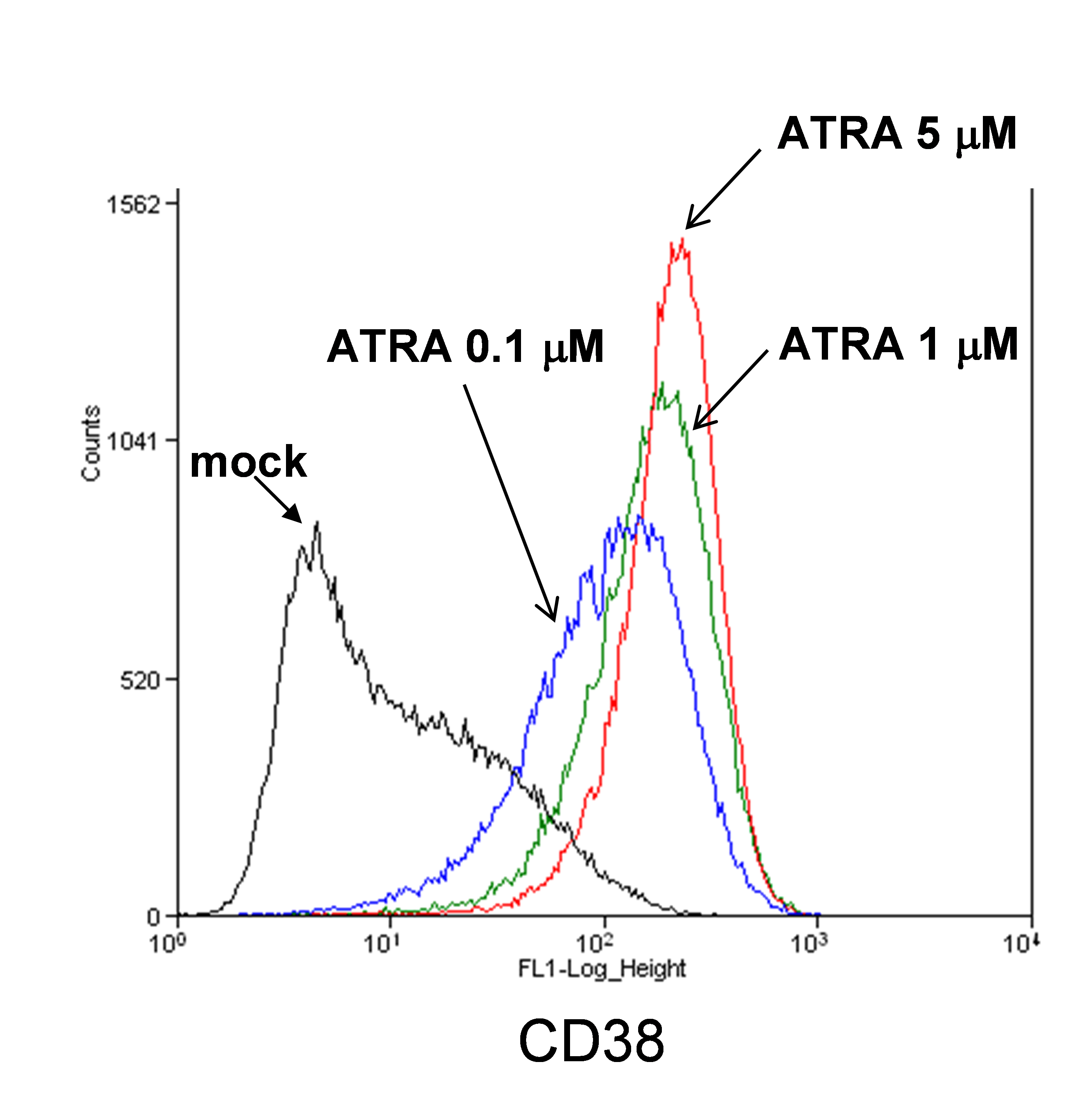

Supplement: Figure S5 — CD38 expression after ATRA treatment. Flow cytometry analysis of CD38 expression in KCL-22 cells treated with ATRA at concentrations indicated. (TIF) [file pgen.1004414.s005.tif]

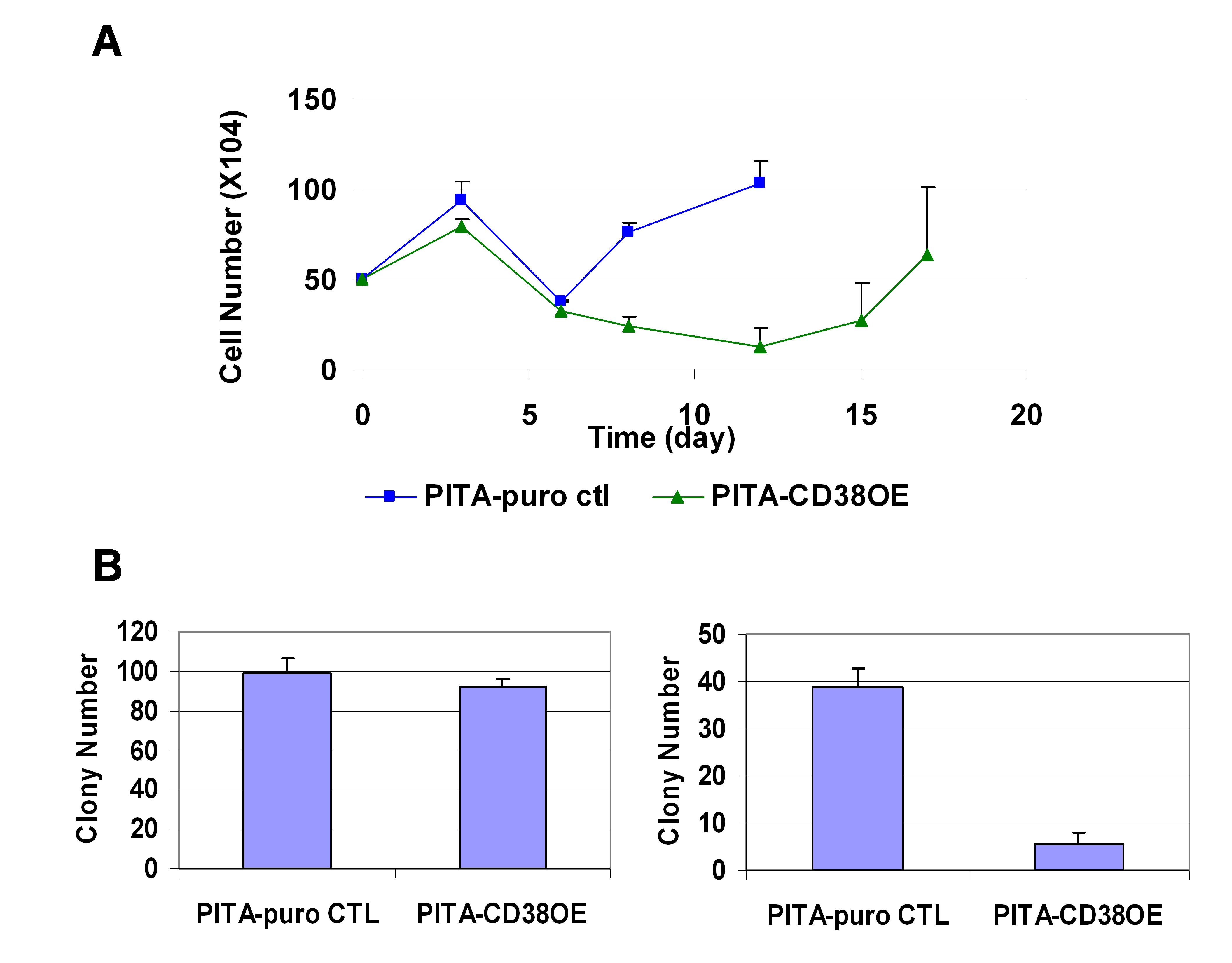

Supplement: Figure S6 — Effect of CD38 expression. Puromycin enriched, CD38 transduced KCL-22 cells (but not sorted) were analyzed for relapse on IM in liquid culture (A) and IM resistant soft agar colony formation (B). In B, left panel was plating efficiency with 500 cells/well in the absence of IM; right panel with 1 million cells/well in 2.5 µM IM. (TIF) [file pgen.1004414.s006.tif]

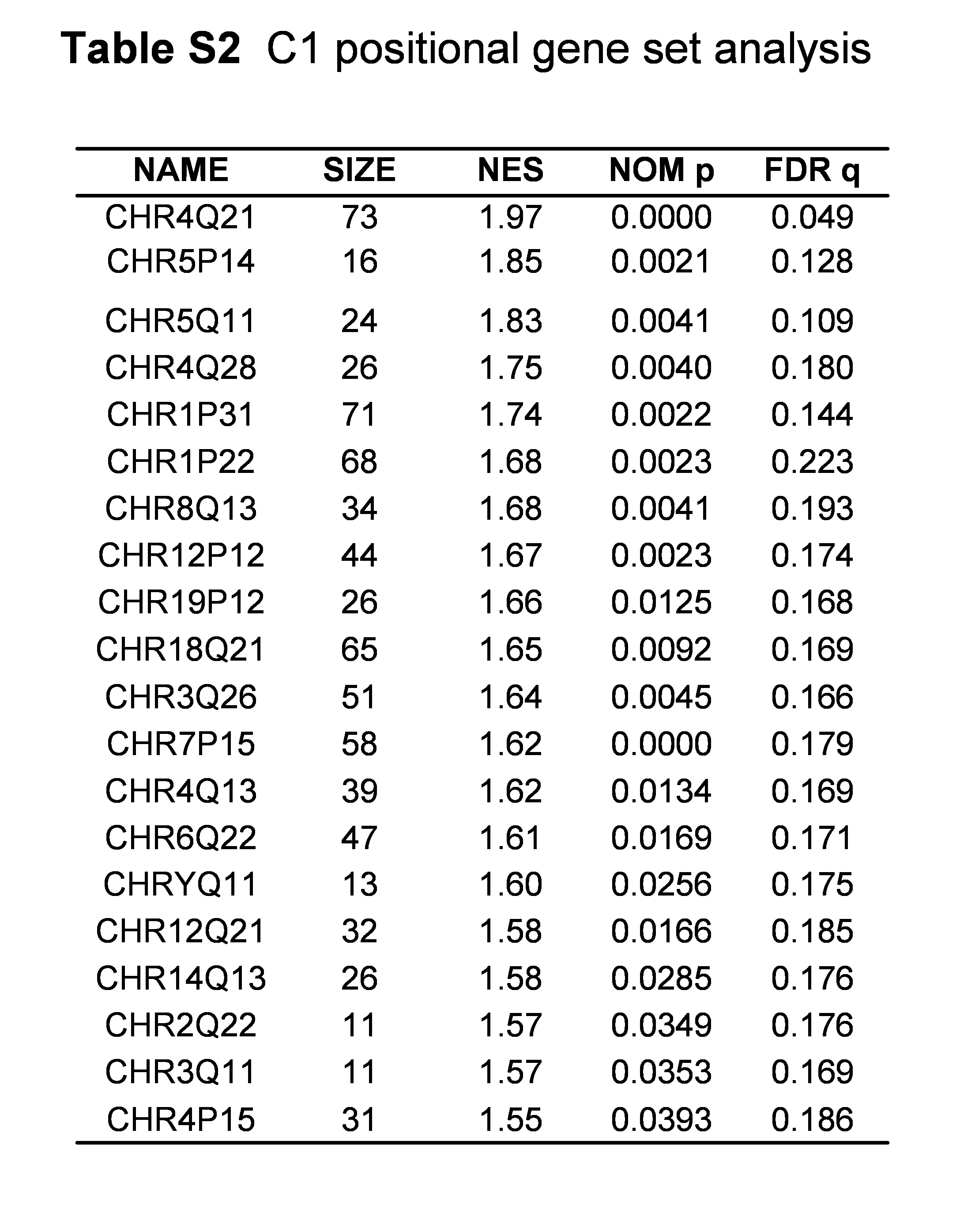

Supplement: Table S2 — C1 positional gene set analysis. (TIF) [file pgen.1004414.s008.tif]

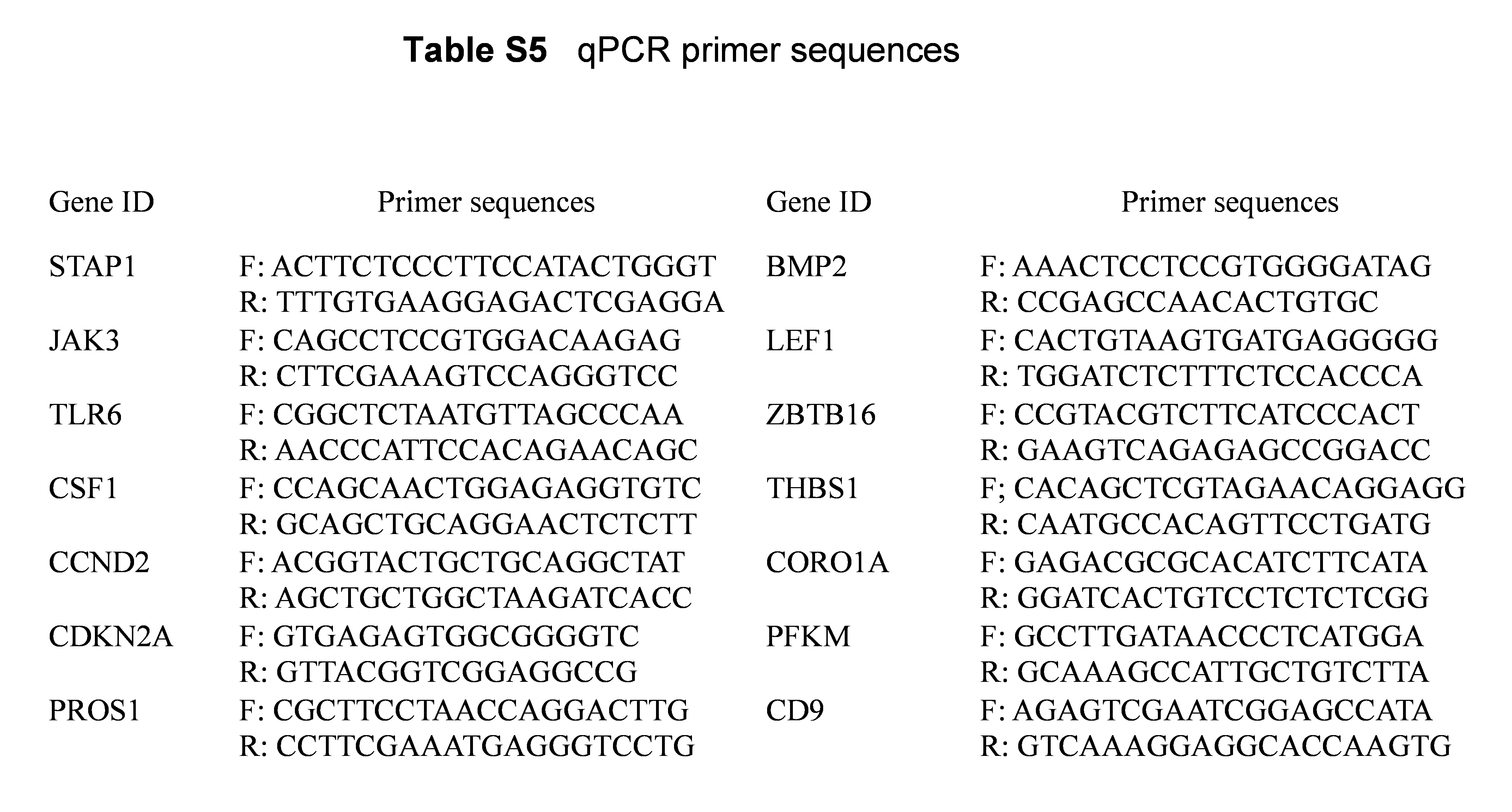

Supplement: Table S5 — qPCR primer sequences. (TIF) [file pgen.1004414.s011.tif]
